# Multi-domain cognitive assessment of male mice shows space radiation is not harmful to high-level cognition and actually improves pattern separation

**DOI:** 10.1038/s41598-020-59419-z

**Published:** 2020-02-17

**Authors:** Cody W. Whoolery, Sanghee Yun, Ryan P. Reynolds, Melanie J. Lucero, Ivan Soler, Fionya H. Tran, Naoki Ito, Rachel L. Redfield, Devon R. Richardson, Hung-ying Shih, Phillip D. Rivera, Benjamin P. C. Chen, Shari G. Birnbaum, Ann M. Stowe, Amelia J. Eisch

**Affiliations:** 1 Department of Psychiatry, University of Texas Southwestern Medical Center, Dallas, TX USA; 2 Department of Anesthesiology and Critical Care Medicine, Children’s Hospital of Philadelphia, Philadelphia, PA USA; 3 Department of Anesthesiology and Critical Care Medicine, Perelman School of Medicine, University of Pennsylvania, Philadelphia, PA USA; 4 Department of Radiation Oncology, University of Texas Southwestern Medical Center, Dallas, TX USA; 5 Department of Neurology and Neurotherapeutics, UT Southwestern Medical Center, Dallas, TX USA; 6 Department of Neuroscience and Mahoney Institute for Neurosciences, Perelman School of Medicine, University of Pennsylvania, Philadelphia, PA USA; 7 Present Address: Oriental Medicine Research Center, Kitasato University, Tokyo, Japan; 8 Present Address: Department of Biology, Hope College, Holland, MI USA; 9 Present Address: Department of Neurology, University of Kentucky, Lexington, KY USA

**Keywords:** Neuroscience, Stem cells

## Abstract

Astronauts on interplanetary missions - such as to Mars - will be exposed to space radiation, a spectrum of highly-charged, fast-moving particles that includes ^56^Fe and ^28^Si. Earth-based preclinical studies show space radiation decreases rodent performance in low- and some high-level cognitive tasks. Given astronaut use of touchscreen platforms during training and space flight and given the ability of rodent touchscreen tasks to assess functional integrity of brain circuits and multiple cognitive domains in a non-aversive way, here we exposed 6-month-old C57BL/6J male mice to whole-body space radiation and subsequently assessed them on a touchscreen battery. Relative to Sham treatment, ^56^Fe irradiation did not overtly change performance on tasks of visual discrimination, reversal learning, rule-based, or object-spatial paired associates learning, suggesting preserved functional integrity of supporting brain circuits. Surprisingly, ^56^Fe irradiation improved performance on a dentate gyrus-reliant pattern separation task; irradiated mice learned faster and were more accurate than controls. Improved pattern separation performance did not appear to be touchscreen-, radiation particle-, or neurogenesis-dependent, as ^56^Fe and ^28^Si irradiation led to faster context discrimination in a non-touchscreen task and ^56^Fe decreased new dentate gyrus neurons relative to Sham. These data urge revisitation of the broadly-held view that space radiation is detrimental to cognition.

## Introduction

Interplanetary missions - such as to Mars - are a high priority for many space agencies. The crew of future missions will face hazards^1–3^, such as exposure to galactic cosmic radiation^4–7^ a spectrum of low and high-(H) atomic number (Z) and high-energy (E) particles such as ^56^Fe and ^28^Si. Fast-moving HZE particles cannot be effectively blocked by modern spacecraft shielding^8–11^. Therefore, it is concerning that studies with laboratory animals generally conclude HZE particles are detrimental to brain and behavior^12–14^. Such preclinical data suggest HZE particle exposure may be harmful to astronaut cognition and impede mission success.

However, there are reasons to revisit the conclusion that HZE particle exposure is detrimental to cognition. First, age at the time of irradiation matters. Most preclinical data that led to the Probabilistic Risk Assessment of HZE particles being detrimental to cognition were from tests performed on young adult rodents (~2-3 months [mon] at exposure)^14^; in many cases, age at testing was not reported^14^. To more accurately reflect the average age of astronauts, NASA now requires ground-based space studies to be performed in mature animals (~6-mon-old at irradiation)^14–24^. Indeed, some studies now directly compare the cognitive impact of HZE irradiation in early life vs. maturity^25–28^, although the results are mixed. Second, type of behavioral test matters. Recent work with mature rodents shows HZE particle exposure decreases performance in some - but not all - behavioral tests; even tests that engage similar neural circuits produce distinct results^14–20,29^. A potential contribution to these task-dependent discrepancies is task-specific testing environment. In humans (including astronauts), automated computerized cognitive assays help control for the influence of testing environments^30–32^. However, such an approach has not been used to assess cognition in mature rodents after HZE exposure. Third, breadth of testing matters. Preclinical studies on space radiation typically assess one or two cognitive domains^33–35^. In contrast, astronauts repeatedly undergo test batteries - often on a touchscreen platform - to assess integrity of many cognitive domains over time^30,36^. To this end, many aspects of neuroscience have employed rodent touchscreen testing, a platform extensively validated for its ability to provide multidimensional assessment of functional integrity of brain circuits in a highly-sensitive and translationally-relevant way^37–39^. In regard to space radiation, it is known that head-only exposure of young adult rats exposed to protons (a low energy particle) does not change acquisition or reversal learning on a touchscreen line discrimination task^40^. However, given the power of touchscreen testing, it is surprising that it is not known how whole body exposure of mature rodents to HZE particles influences performance on a battery of rodent touchscreen tests. This is particularly notable as the touchscreen platform permits analysis of many higher cognitive functions - such as pattern separation - which are part of the astronaut’s mission-critical skill set yet which have not been preclinically assessed for their sensitivity to space radiation.

To address these major knowledge gaps, mature C57BL/6J male mice received either Sham irradiation (IRR) or whole body ^56^Fe particle IRR and were assessed on a battery of touchscreen cognitive tasks to assess complex learning, cognitive flexibility, visuospatial learning, and stimulus-response habit learning^39,41–43^. This touchscreen battery revealed an unexpected finding: improved pattern separation in ^56^Fe IRR vs. Sham mice. To assess whether this improvement was dependent on touchscreen testing or on ^56^Fe IRR, we then assessed separate cohorts of ^56^Fe IRR vs. Sham mice for pattern separation performance in a non-touchscreen task, contextual discrimination fear conditioning (CDFC), and also assessed the impact of ^28^Si IRR vs. Sham on CDFC. Irrespective of whether tested on a touchscreen or non-touchscreen platform, or whether CDFC mice received to ^56^Fe or ^28^Si IRR, IRR mice had better pattern separation than Sham mice. Taken together, these data show whole body exposure to HZE particles is not detrimental to high level cognition in mature mice and actually enhances performance in certain mission-critical tasks, such as pattern separation.

## Results

### Mice given whole body ^56^Fe IRR demonstrate overall normal perceptual discrimination, association learning, and cognitive flexibility in touchscreen testing

**Figure 1:**
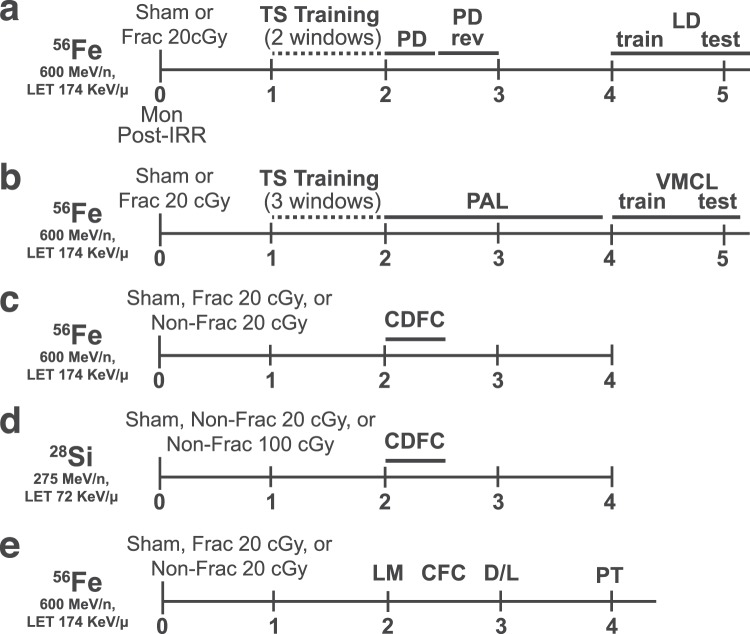
Timeline of experimental groups and behavior tests. **(a-e)** Separate, independent cohorts of C57BL/6J male mice (JAX Cat. #000664) received whole-body exposure to particles of ^56^Fe **(a-c, e)**, ^28^Si **(d)**, or Sham exposure at 6-months (mon) of age (0-mon post-irradiation [IRR]). **(a)** ^56^Fe or Sham mice subsequently were run on TS training, PD, PD rev, and LD. **(b)** ^56^Fe or Sham mice were run on TS training, PAL, and VMCL. **(c)** ^56^Fe or Sham mice were run on CDFC. **(d)** ^28^Si or Sham mice were run on CDFC. **(e)** ^56^Fe or Sham mice were run on LM, CFC, D/L, and PT, and brains were collected for DCX + cell quantification. For each set of mice shown **(a-e)**, the interval between radiation exposure and behavioral testing was equal between Sham and IRR groups. Specifically, the beginning of each major behavioral test shown above was synchronized in Sham and IRR cohorts. CDFC = contextual discrimination fear conditioning, CFC = contextual fear conditioning, D/L = dark/light box test, Frac = fractionated, IRR = irradiation, LD = location discrimination, LM = locomotor, mon = months, Non-Frac = non-fractionated, PAL = paired associates learning, PD = pairwise discrimination, PD rev = PD reversal, PT = pain threshold, TS = touchscreen, VMCL = visuomotor conditioning learning.

Six-mon-old male C57BL/6J mice received either Sham IRR or fractioned (Frac) whole body 20 cGy ^56^Fe (3 exposures of 6.7 cGy every-other day, total 20 cGy) **(**Figs. 1a,c,e**)**. This total dose is submaximal to that predicted for a Mars mission^44,45^, and the fractionation interval (48 hours [hr]) was determined by the inter-fraction period for potential repair processes^46^. As previously reported^47^, this dose and these fractionation parameters do not interfere with weight gain or cause hair loss (Fig. S1a).

**Figure 2:**
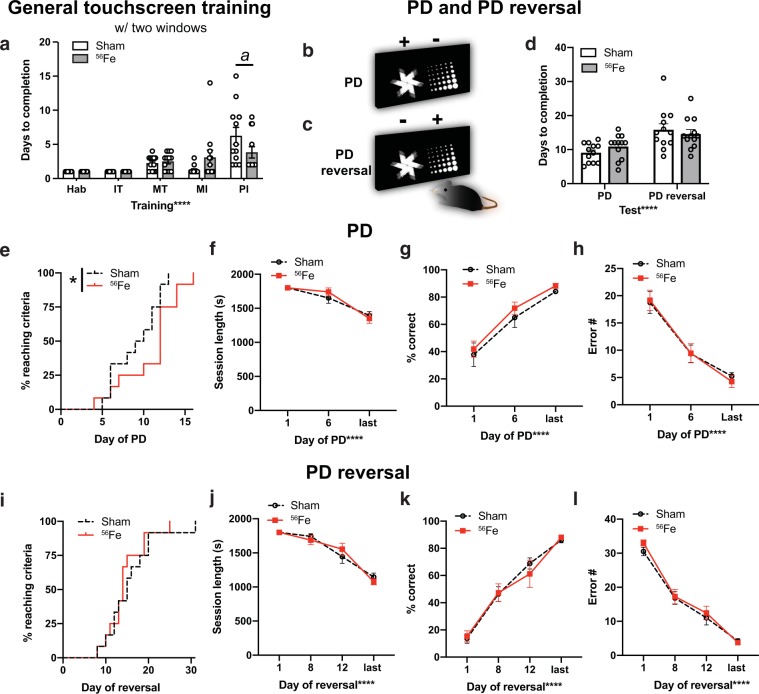
Mice exposed to ^56^Fe IRR at 6-month of age complete the final stage of general touchscreen training in fewer days compared to Sham mice, but perform similarly to Sham mice overall in the Pairwise Discrimination (PD) and reversal (PD rev). **(a)** Sham and ^56^Fe IRR mice performed similarly in the first four steps of general touchscreen training with two windows: Habituation (Hab), Initiate Touch (IT), Must Touch (MT), and Must Initiate (MI). However, ^56^Fe IRR mice completed the Punish Incorrect (PI) stage of general touchscreen training in fewer days than Sham mice. **(b-c)** Sample touchscreen images for PD and PD reversal tests. **(d)** Sham and ^56^Fe IRR mice completed PD and PD rev in similar number of days. **(e)** Cumulative distribution function showing the difference in the rate of days required to complete PD between Sham and ^56^Fe IRR mice. **(f–h)** Sham and ^56^Fe IRR mice performed similarly in PD. (**f**) session length, (**g**) percent (%) correct, (**h**) Error number (#). **(i)** Cumulative distribution function showing no difference in the test days required to complete PD rev between two groups. **(j–l)** Sham and ^56^Fe IRR mice performed similarly in PD rev. (**j**) Session length, (**k**) % correct, (**l**) Error #. Sham: n = 12, IRR: n = 12. Mean ± SEM. Two-way RM ANOVA in **a,d,f–h,j–l**, *p < 0.05, ****p < 0.0001, post hoc: Bonferroni *a* p < 0.05 in Sham vs. ^56^Fe; Mantel-Cox test in **e,i**, *p < 0.05. s = seconds.

Beginning 1 mon post-IRR, Sham and ^56^Fe IRR mice began training on a touchscreen platform extensively validated in rodents^39,41,43,48,49^**(**Fig. 1a,b**)**. Mice initially went through five stages of general touchscreen training **(**Fig. 2a**)**, with performance reflecting instrumental or operant learning. Sham and ^56^Fe IRR mice completed most stages of the initial operant touchscreen training in similar periods of time (Fig. 2a). The exception was the final stage, Punish Incorrect (PI, where an incorrect trial to timeout); on average, ^56^Fe IRR mice finished PI in ~40% fewer days versus Sham mice (Fig. 2a, Table S1).

Mice then advanced to pairwise discrimination (PD, visual discrimination) and PD reversal (reversal learning, Fig. 2b,c), tests which reflect perceptual discrimination and association learning as well as cognitive flexibility, respectively, and rely on cortical (prefrontal, orbital frontal, perirhinal) and striatal circuits^41,48^. On average, both ^56^Fe IRR and Sham mice completed PD and PD reversal in a similar number of days (Fig. 2d, Table S1). Analysis of the distribution of subjects to reach criteria each day revealed significant difference between Sham and ^56^Fe IRR mice **(**Fig. 2e**)**. Specifically, 50% of Sham mice reached PD completion criteria at 9.5 days, while 50% ^56^Fe IRR mice reached criteria at 12 days. However, Sham and ^56^Fe IRR mice did not differ with regard to average session length, percent correct, or number of errors **(**Fig. 2f,h**)**. In PD reversal, the distribution of subjects to reach completion criteria was not different between Sham and ^56^Fe IRR mice **(**Fig. 2i**)**, with 50% of Sham and ^56^Fe IRR mice reaching PD reversal completion criteria at 15 and 14 days, respectively **(**Fig. 2i**)**. As with PD, Sham and ^56^Fe IRR mice did not differ in PD reversal average session length, percent correct, or number of errors **(**Fig. 2j,l**)**.

### Mice given whole body ^56^Fe IRR demonstrate normal visuospatial learning and stimulus-response habit learning in touchscreen testing

**Figure 3:**
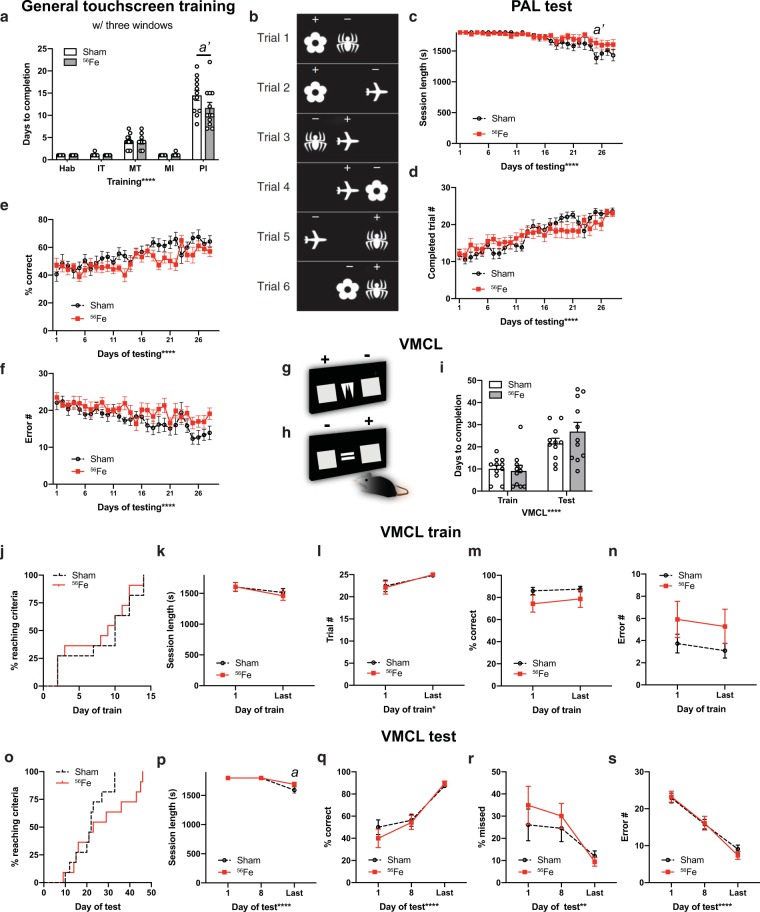
Mice exposed to ^56^Fe IRR at 6-month of age complete the final stage of general touchscreen testing in fewer days than Sham mice, but perform similarly to Sham in tests of rule-based learning and stimulus-response habit learning. **(a)** Sham and ^56^Fe IRR mice performed similarly in the 4 first steps of general touchscreen training stages with three windows, including Habituation (Hab), Initiate Touch (IT), Must Touch (MT), and Must Initiate (MI). However, ^56^Fe IRR mice completed the Punish Incorrect (PI) stage of general touchscreen training in fewer days than Sham mice. **(b)** Sample touchscreen images for Paired Associates Learning (PAL). **(c–f)** Sham and ^56^Fe IRR mice performed similarly in PAL. (**c**) session length, (**d**) completed trials, (**e**) percent (%) correct, (**f**) Error number (#). **(g,h)** Sample touchscreen images for Visuomotor Conditional Learning (VMCL) train and test phases. **(i)** Sham and ^56^Fe IRR mice performed similarly in VMCL train and test. **(j)** Cumulative distribution function showed no difference in days required to complete training. Distribution of Sham and ^56^Fe IRR mice (n = 11/group) did not differ in days required to complete VMCL training. **(k–n)** Sham and ^56^Fe IRR mice performed similarly in VMCL train. (**k**) session length, (**l**) completed trials, (**m**) % correct, (**n**) Error #. **(o)** Cumulative distribution function showed no difference in days required to complete VMCL test. Distribution of Sham and ^56^Fe IRR mice (n = 11/group) did not differ in VMCLtest. **(p–s)** Sham and ^56^Fe IRR mice performed similarly in VMCL test. (**p**) Session length, (**q**) % correct, **(r)** % missed, (**s**) Error #. Sham: n = 12 **(a–f)**, 11 **(i–s)**, IRR: 12 **(a–f)**, 11 **(i–s)**. Mean ± SEM. Two-way RM ANOVA in **a,c–f,i,k–n,p–s**, *p < 0.05, **p < 0.001, ****p < 0.0001, post hoc: Bonferroni *a* p < 0.05, *a’* p < 0.01 in Sham vs. ^56^Fe; Mantel-Cox test in **j,o**.

A parallel group of mice was used to assess the influence of ^56^Fe IRR object-location paired associates learning (PAL) and visuomotor conditional learning (VMCL) which reflect visuospatial and stimulus-response habit learning, respectively, and rely on intact circuits of the hippocampus^41,43^ (PAL) and striatum and posterior cingulate cortex^41,43^ (VMCL, Fig. 1b**)**. Consistent with results in the first cohort of mice, Sham and ^56^Fe IRR mice completed most stages of operant touchscreen training in similar periods of time (Fig. 3a), again with the exception of PI where ^56^Fe IRR mice finished in ~20% fewer days than Sham.

In PAL (Fig. 3b), Sham and ^56^Fe IRR mice were similar in session length, number of trials, percent correct, and number of errors over the 29-day testing period **(**Fig. 3c-f**)**. In both VMCL train and test (Fig. 3g,h), Sham and ^56^Fe IRR mice had similar average days to completion **(**Fig. 3i**)**. In VMCL train, Sham and ^56^Fe IRR mice performed similarly in regard to distribution of subjects to reach criteria each training day (50% subjects reached criteria at 10 days in Sham mice vs. 9 days in ^56^Fe IRR mice), session length, number of trials, percent correct, and number of errors (VMCL train; Fig. 3j-n, Table S1). In VMCL test, Sham and ^56^Fe IRR mice had similar distribution of subjects to reach criteria each training day (50% of subjects reached criteria at 22 days in Sham mice vs. 23 days in ^56^Fe IRR mice), session lengthpercent correct, percent missed, and number of errors (VMCL test; Figs. 3o-s, Table S1). However, the time to complete the session on the last day of VMCL test was longer in ^56^Fe IRR relative to Sham mice (Fig. 3p).

### Whole body ^56^Fe IRR improves pattern separation in an appetitive-based location discrimination touchscreen task

**Figure 4:**
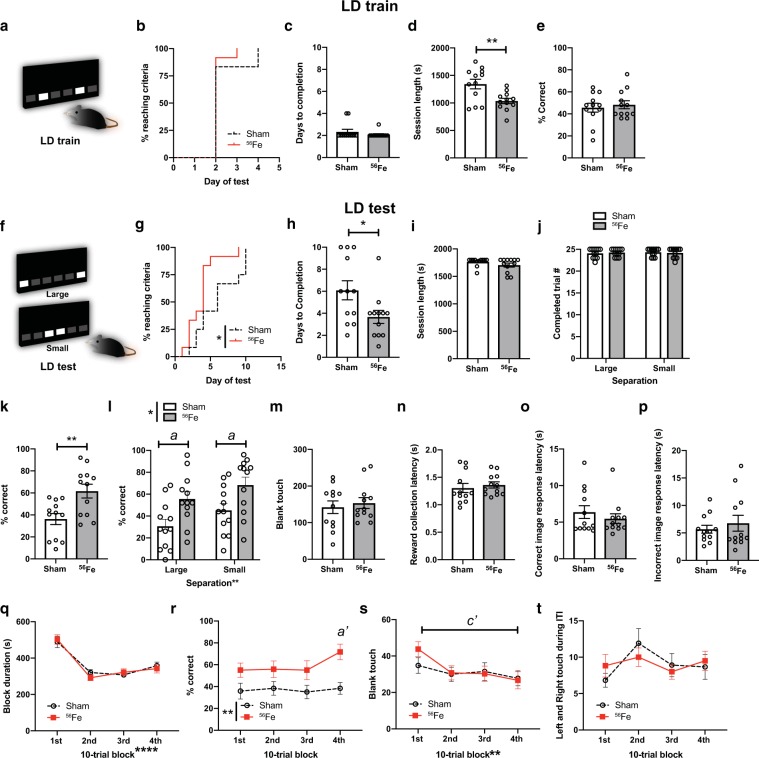
On an appetitive pattern separation task, mice exposed to ^56^Fe IRR at 6-month of age distinguish two similar visual cues earlier and with greater accuracy on the last test day relative to Sham mice. **(a)** Sample touchscreen images for location discrimination training (LD train). **(b–e)** Sham and ^56^Fe IRR mice performed similarly in LD train. (**b**) Distribution of subjects reaching criteria, (**c**) days to completion, (**d**) session completion time, (**e**) % correct. **(f)** Sample touchscreen images for LD testing (LD test). **(g–j)** ^56^Fe IRR mice completed the LD test earlier than Sham **(g,h)**, but no difference in session completion time **(i)** or number of completed trials **(j)**. **(k,l)** ^56^Fe IRR mice were more accurate overall **(k)** and on both “Large” and “Small” separation trials compared to Sham mice **(l)**. **(m–p)** Sham and ^56^Fe IRR mice made similar number of blank touches to non-stimuli windows **(m)** and had similar reward collection latency **(n)**, correct image response latency **(o)**, and incorrect image response latency **(p)**. **(q–t)** Sham and ^56^Fe IRR mice had similar block duration in each 10-trial block **(q)**. However, ^56^Fe IRR mice had higher accuracy in the 4^th^ 10-trial block (31^st^–40^th^ trial) compared to Sham mice **(r)**. Sham and ^56^Fe IRR mice made similar number of blank touches in each block **(s)** and left and right touches during inter-trial interval (ITI) **(t)**. Sham: n = 12, IRR: n = 12. Mean ± SEM. Mantel-Cox test, *p < 0.05 in **b, g**; Unpaired, two-tailed t-test in **c-e, h-i, k, m-p**; Two-way RM ANOVA,*p < 0.05, **p < 0.01, post hoc: Bonferroni in **j, l, q-t**, *a* p < 0.05, *a’* p < 0.01 in Sham vs.^56^Fe mice in **l, r**, *c*’ p < 0.01 1^st^ and 4^th^ block in ^56^Fe mice in s. s = seconds.

A brain region commonly studied with regard to space radiation-induced deficits in function and activity-dependent processes (i.e. neurogenesis) is the hippocampal dentate gyrus^47,50–52^. Based on prior work, we hypothesized whole body ^56^Fe IRR impairs pattern separation, a cognitive function reliant on dentate gyrus integrity^53,54^. To test this hypothesis, Sham and ^56^Fe IRR mice were assessed on a touchscreen pattern separation task: location discrimination (LD)^39^**(**Fig. 1a**)**. In the LD training portion of the assessment (LD train, Fig. 4a), Sham and ^56^Fe IRR mice had similar distribution of the proportion of subjects reaching criteria **(**Fig. 4b**)**, average days to completion, session length, and percent correct **(**Fig. 4c,e**)**. However, Sham and ^56^Fe IRR mice differed in LD performance (LD test, Fig. 4f) in several aspects. First, the distribution of proportion of subjects reaching criteria was distinct in ^56^Fe IRR mice vs. Sham mice **(**Fig. 4g**)**. ^56^Fe IRR mice reached criteria at >3x faster rate vs. Sham mice, and 50% of ^56^Fe IRR mice reached criteria by 4 days versus Sham mice reaching criteria by 6 days. Second, ^56^Fe IRR mice completed LD test in fewer days than Sham mice **(**Fig. 4h**)**, although both groups showed similar session length and number of completed trials **(**Fig. 4i,j**)**. Third, ^56^Fe IRR mice performed LD test more accurately than Sham mice both overall **(**Fig. 4k**)** as well when presented with stimuli separated by either large or small distances **(**Fig. 4l**)**.

We next behaviorally-probed reasons why ^56^Fe IRR mice had improved pattern separation relative to Sham mice. For example, the improved location discrimination in ^56^Fe IRR mice may be reflective of unintentional screen touches, perhaps due to IRR-induced alteration of attention to stimuli or motivation to obtain reward. However, the number of blank touches **(**Fig. 4m**)**, reward collection latency (Fig. 4n), and choice latency (Fig. 4o,p) were not different between ^56^Fe IRR mice and Sham mice. Also, since the location of the rewarded stimuli changed daily but maintained within each session, it is possible that pattern separation is progressively improved within a session, particularly on the last test day. Sham and ^56^Fe IRR mice had similar last day block duration and left/right touches during intertrial interval, but ^56^Fe IRR mice had a greater percent correct during the 4th 10-trial block relative to Sham mice **(**Fig. 4q,r,t**)**. In addition, while Sham mice did not differ between the 1st and 4th 10-trial blocks on the last day, ^56^Fe IRR mice had fewer blank touches in the 4th 10-trial block relative to the 1st 10-trial block. These data suggest that on the last day of LD, ^56^Fe IRR mice demonstrate within-session enhanced pattern separation **(**Fig. 4s**)**.

### Whole body ^56^Fe and ^28^Si IRR improves pattern separation in a foot shock- based contextual discrimination task

To assess whether ^56^Fe IRR-induced improvement in pattern separation was restricted to appetitive tasks, a parallel cohort of mice was exposed to Sham or ^56^Fe IRR and tested on pattern separation using a classic pattern separation behavior paradigm: contextual discrimination fear conditioning (CDFC)^54,55^. To specifically assess whether particle inter-fraction interval influenced behavioral outcome, 6-mon-old C57BL/6J mice received Sham IRR, whole body fractionated 20 cGy (Frac 20 cGy; 3 exposures of 6.7 cGy) ^56^Fe IRR, or whole body non-fractionated 20 cGy (Non-Frac 20 cGy; 1 exposure of 20 cGy) ^56^Fe IRR **(**Fig. 1c**)**. As previously reported^47^, Sham IRR, Frac 20 cGy, and Non-Frac 20 cGy mice had similar weight changes over time (Fig. S1a).

**Figure 5:**
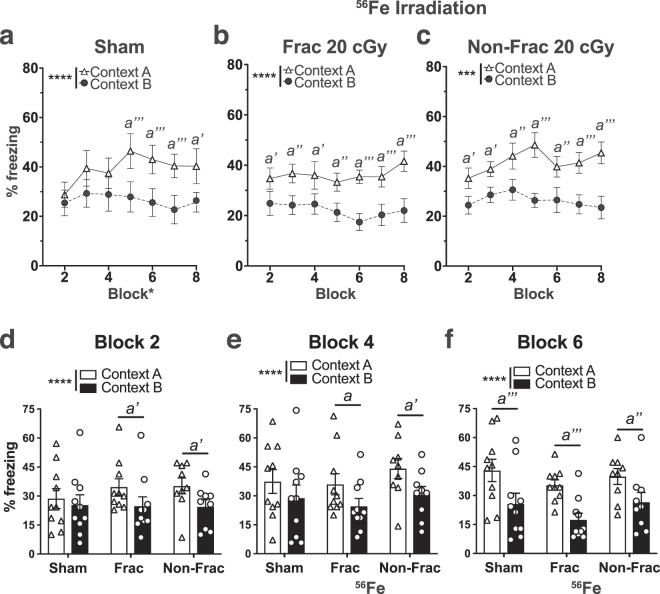
On an aversive pattern separation test, mice exposed to whole body ^56^Fe IRR at 6-month of age discriminate two contexts earlier than mice exposed to Sham IRR. **(a)** Sham mice discriminate Context A (shock context) from Context B (non-shock context) by Block 5. **(b**,**c)** Frac **(b)** and Non-Frac **(c)** ^56^Fe mice discriminate Context A from Context B by Block 2. **(d–f)** When examined at Block 2 **(d)**, Block 4 **(e)**, and Block 6 **(f)**, Frac and Non-Frac ^56^Fe discriminate by Block 2. Sham: n = 10, Frac:n = 10, Non-Frac: n = 9. Mean ± SEM. Two-way RM ANOVA, *p < 0.05, **p < 0.01, ***p < 0.001, ****p < 0.0001, Bonferroni post-hoc tests in **a**–**f**. *a* p < 0.05, *a’* p < 0.01, *a”* p < 0.001, *a”’* p < 0.0001 in Context A vs B. Frac = fractionated, Non-Frac = non-fractionated.

Beginning ~2-mon post-IRR (8 mon of age), mice underwent CDFC (Figs. 5, S2) to learn that one context (Context A) was paired with a foot shock while another similar context (Context B) was a non-shock context. When tested in CDFC, Sham mice discriminated the two contexts by Days 9–10 (Block 5), as they froze more in the shock-paired context (Context A) compared to the non-shock context (Context B; Fig. 5a, Table S1). However, mice exposed to either Frac 20 cGy or Non-Frac 20 cGy of ^56^Fe IRR discriminated the contexts by Days 3–4 (Block 2, Fig. 5b,c, Table S1). Direct comparison across treatment groups revealed Frac 20 cGy and Non-Frac 20 cGy mice froze more in Context A vs. Context B in Blocks 2 and 4, earlier than Sham (Fig. 5d,f, Table S1). Possible explanations for these results include differential activity, anxiety, or pain sensitivity in Sham vs. ^56^Fe IRR mice. To address these possibilities, parallel groups of mice underwent assessment for locomotion (Fig. S1b), dark/light testing (Fig. S1c,d) and pain threshold (Fig. S1e,g). However, Sham, Frac, and Non-Frac mice performed similarly on all these tests (Fig. S1b,g**)**. Thus, both Frac and Non-Frac 20 cGy ^56^Fe IRR mice learned to pattern separate earlier relative to Sham mice without overt changes in locomotion, anxiety-like behavior, or sensitivity to pain.

**Figure 6:**
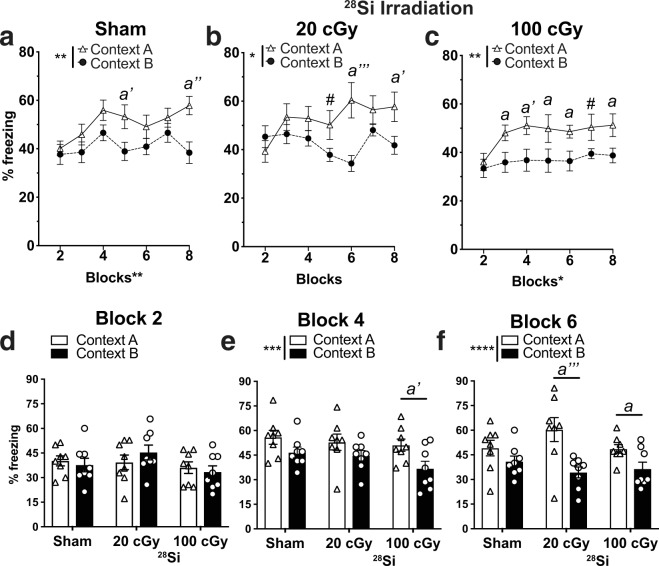
On an aversive pattern separation test, mice exposed to a different HZE particle - ^28^Si - at 6 month of age also discriminate two contexts earlier than mice exposed to Sham IRR. **(a)** Sham mice discriminate Context A (shock context) from Context B (non-shock context) by Block 5. **(b,c)** While 20 cGy ^28^Si mice **(b)** discriminate Context A from Context B by Block 5, 100 cGy ^28^Si mice **(c)** discriminate by Block 3. **(d–f)** When examined at Block 2 **(d)**, Block 4 **(e)**, and Block 6 **(f)**, 100 cGy Si mice by Block 4 and both 20 cGy and 100 cGy ^28^Si mice discriminate by Block 6. Sham: n = 8, Frac:n = 8, Non-Frac: n = 8. Mean ± SEM. Two-way RM ANOVA, *p < 0.05, **p < 0.01, ***p < 0.001, ****p < 0.0001, Bonferroni post-hoc tests in **a-c,e-f**, #p = 0.05–0.06, *a* p < 0.05, *a’* p < 0.01, *a”* p < 0.001, *a”’* p < 0.0001 in Context A vs. B.

To determine if the improvement in CDFC pattern separation generalized to other fear-based hippocampal- and amygdala-based learning, a parallel cohort of mice received Sham or ^56^Fe IRR and underwent classical contextual fear conditioning (CFC; Fig. 1e, Fig. S3a,b). Sham and ^56^Fe IRR mice (both Frac and Non-Frac 20 cGy groups) performed similarly in the context test (Fig. S3c) and in the cue test both pre-tone and during tone (Fig. S3d). Importantly, to see if the space radiation-induced improvement in CDFC was dependent on the type of heavy particle used, CDFC was also performed with mice exposed to whole body ^28^Si IRR (Figs. 1d and 6), a particle with a smaller track structure than ^56^Fe^56^. Sham mice spent more time freezing in Context A vs. Context B only on Days 9–10 (Block 5) and Days 15–16 (Block 8, Figs. 6a). Mice exposed to 20 cGy of ^28^Si discriminated between the two contexts as early as Days 11–12 (Block 6; Fig. 6bf). Notably, mice exposed to 100 cGy of ^28^Si were able to discriminate between the two contexts as early as Days 5–6 (Block 3; Fig. 6ce,f). Taken together, these data show that exposure to two different HZE particles - either ^56^Fe or ^28^Si - results in earlier separation ability relative to Sham mice on the shock-based CDFC pattern separation test.

### ^56^Fe IRR decreases dentate gyrus neurogenesis 4 mon post-IRR

**Figure 7:**
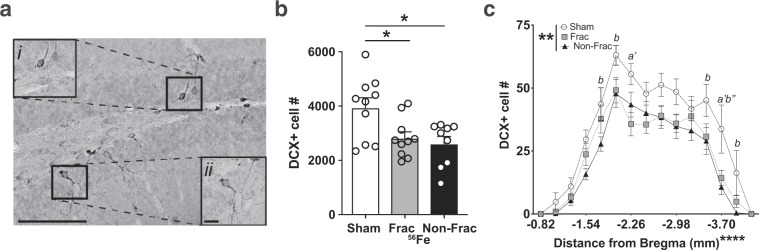
Stereological quantification reveals fewer immature dentate gyrus neurons (doublecortin (DCX)+ cells) 4 months post-whole body ^56^Fe particle IRR relative to Sham mice. **(a)** Representative photomicrograph of DCX+ cell in the mouse dentate gyrus subgranular zone. Insets: higher magnification of boxed areas in main image. Scale bar = 100 um in **a**, 10 um in inset ii. **(b**,**c)** Relative to Sham mice, Frac, and Non-Frac ^56^Fe mice have fewer DCX + dentate gyrus cells. Sham: n = 10, Frac:n = 10, Non-Frac: n = 9. Mean ± SEM. One-way ANOVA Bonferroni posthoc. *p < 0.05 in **b**, Two-way ANOVA, Bonferroni posthoc. *a’* p < 0.01 Sham vs. Frac, *b* p < 0.05, *b”* p < 0.001 Sham vs. Frac in **c**. Frac = fractionated, Non-Frac = non-fractionated.

Pattern separation ability is dependent on new dentate gyrus neurons as well as dentate gyrus activity, and an inducible increase in adult neurogenesis improves pattern separation^54,57,58^. To assess whether the IRR-induced improvement in pattern separation reported here was correlated with increased neurogenesis, we used stereology to quantify the number of cells in the dentate gyrus immunoreactive for doublecortin (DCX, Fig. 7a), a widely-accepted marker for neurogenesis^59^. Although mice exposed to either Frac or Non-Frac ^56^Fe IRR had improved context discrimination compared to control mice **(**Fig. 5), these mice had fewer DCX + cells compared to control mice (Fig. 7b,c, Table S1).

## Discussion

Astronaut training and in-mission assessment rely on touchscreen testing due to its flexibility in probing a variety of cognitive functions. Rodent touchscreen testing similarly allows researchers to probe the multidimensional functional integrity of brain circuits in a highly-sensitive and translationally-relevant way^37,39,41,60^, but prior to the present work it was unknown how exposure to the HZE particles that comprise space radiation influences touchscreen performance. Based on the large literature with young animals and the negative impact of HZE particle exposure on the central nervous system^13,61^, we hypothesized whole-body exposure to ground-based HZE particles would diminish the performance of mice in touchscreen-based behaviors, particularly those behaviors reliant on the dentate gyrus, such as pattern separation. The results of our multi-domain cognitive assessment showed our hypothesis was wrong. Mature mice exposed to either Sham IRR or HZE particles performed similarly in touchscreen tasks of visual discrimination, cognitive flexibility, rule-based learning, and object-spatial associated learning, in classical hippocampal- and amygdala-based tasks (i.e. CFC), and in tasks that detect anxiety-like behavior (i.e. D/L). Surprisingly, IRR mice performed better than Sham IRR mice in pattern separation tasks when assessed on either appetitive (LD test) or aversive (CDFC) platforms. Both ^56^Fe and ^28^Si IRR resulted in earlier and more consistent pattern separation in CDFC vs. respective Sham groups, showing that HZE-induced improvement is not specific to a particular HZE particle. While this study was not powered to assess dose-dependence, 20 cGy ^56^Fe IRR (either fractionated or non-fractiationed) appeared to improve pattern separation in CDFC more effectively than 20 cGy of ^28^Si and roughly similar to 100 cGy of ^28^Si, a result which merits additional future study. Taken together, our study suggests whole body exposure of HZE particles in maturity is not detrimental to high-level cognition, and actually enhances performance specifically in the mission-critical task of pattern separation.

There are three aspects of the present results that are notable from the perspective of behavioral neuroscience in general, and multiple memory systems in particular^62,63^. First, in both humans and rodents, hippocampal damage can actually facilitate behavioral performance on certain tasks^64,65^. For example, when amnesic patients with partial hippocampal injury are given extended exposure to study materials, they can improve their recognition memory to the level of control subjects. Such an improvement is not seen after severe hippocampal injury. Thus, it is reasonable to consider whether the improved pattern separation ability presented here result from HZE particle-induced partial damage to the hippocampus. This is unlikely, as the HZE particle parameters used here do not induce detectable damage to post-mitotic neurons in the adult rodent brain^66,67^ or, as shown here, deficits in other tasks that engage the hippocampus (PAL, CFC). Second, as memory mechanisms in the medial temporal lobe (i.e. hippocampus) and basal ganglia (i.e. dorsal striatum) may sometimes compete^63^, it is possible the improved dentate gyrus-based pattern separation reported here is associated with decreased dorsal striatum-based ‘habit’ learning. However, we find pattern separation is improved in ^56^Fe relative to Sham mice without a change in VMCL habit learning, suggesting normal dorsal striatal function. Finally, the improved pattern separation reported here is reminiscent of the excessive attention seen in some psychiatric disorders - such as autism or obsessive compulsive disorder (OCD) - and in animal models for these disorders^68,69^. Evaluation of autistic- or OCD-like behavioral patterns after HZE particle exposure using other touchscreen paradigms (i.e. extinction, 5-choice serial reaction time test, 5-choice continuous performance reaction task) would clarify whether the improved pattern separation ability demonstrated here is accompanied by maladaptive behaviors (i.e. impaired attention and increased impulsivity)^70,71^.

What might be the neural mechanism underlying the improved pattern separation in HZE-irradiated mice reported here? One possibility is an HZE-induced shift in underlying brain circuit activity. In rodents and humans, pattern separation requires the appropriate balance of activity in the entorhinal cortex-dentate gyrus network^57,72–74^. In aged humans, a decline in pattern separation is proposed to be due to a hypoactive anterolateral enthorhinal cortex and hyperactive dentate gyrus/CA3^75^. Thus, it is possible the HZE-induced improved pattern separation reported here in mouse results from an opposite activity shift: a hyperactive enthorhinal cortex and hypoactive dentate gyrus/CA3. Indeed, in rodents, pattern separation performance is correlated with dentate gyrus activity; better performance results in a hypoactive dentate gyrus, and worse performance results in a hyperactive dentate gyrus^54,72^. As pattern separation engages distinct hippocampal networks relative to other hippocampal-dependent tests (such as novel object recognition)^76,77^, such an HZE-induced shift in hippocampal networks may explain why we see improved pattern separation - while other groups see decreased novel object recognition - after HZE exposure.

Another possibility is that the improved pattern separation we report in HZE-irradiated mice is due to HZE-induced conditions in the dentate gyrus that favor “sparse encoding” of entorhinal cortical input. Sparse encoding is the concept that information - a stimulus, context, experience, memory, etc. - is represented by a relatively small number of simultaneously-active neurons^78^. Sparse encoding in dentate gyrus granule cell neurons is critical for pattern separation, as it minimizes interference between memory representations of similar but not identical experiences^79–83^. This sparsity is due in part to inhibition of dentate gyrus granule cell neurons by GABAergic interneurons and mossy cells^84,85^. It is unknown how the HZE particle parameters used here influence dentate gyrus GABAergic interneurons and mossy cells in mature mice. However, exposure to other energetic particles that comprise space radiation alters the inhibitory network in the dentate gyrus and other hippocampal subregions of young adult rodents^86,87^. In the future, evaluation of GABAergic signaling and other measures relevant to sparse encoding (e.g. number and functionality of hilar interneurons and mossy cells, pattern of memory-induced immediate early gene activation) after Mars-relevant exposure to space radiation would allow testing of the hypothesis that HZE-induced improvement in sparse encoding contributes to the HZE-induced improvement in pattern separation reported here.

A third possibility - and related to conditions that favor sparse encoding - is that HZE particle exposure increases dentate gyrus neurogenesis. In young adult rodents, inducible increase in hippocampal neurogenesis improves pattern separation, while inducible decrease in neurogenesis impairs pattern separation^54,58,88^. However, here we show that improved pattern separation is not correlated with the number of new hippocampal neurons, at least when examined 4 mon-post IRR (when the touchscreen pattern separation testing began in a parallel group of mice). The present study did not assess the number of new neurons 2 mon-post IRR (when CDFC testing began), and did not assess if IRR influences other measures of neurogenesis, such as synaptic connectivity and dendritic integration. However, these data add to the growing evidence that the number of new neurons does not always predict pattern separation performance, particularly in older rodents^89,90^. In fact, decreased hippocampal neurogenesis is proposed to diminish sensitivity to memory interference and thus improve performance in certain memory tasks^90,91^. Computational models support that decreased neurogenesis may enhance sparse encoding^92,93^, which as mentioned above may explain why we see improved pattern separation after HZE particle exposure yet other groups see decreased performance in their behavioral tests.

The disconnect shown here between pattern separation and hippocampal neurogenesis raises interesting future directions. Although historically tied to learning and memory, hippocampal neurogenesis also plays a role in forgetting^94^ with high levels of hippocampal neurogenesis facilitating the forgetting of prior memories, resulting in greater cognitive flexibility^95^. In converse, lower levels of hippocampal neurogenesis - as seen with age - facilitate the persistence of prior memories, lead to more interference with new memory formation, and thus may decrease cognitive flexibility^95^. As here we show irradiated mice have decreased neurogenesis relative to control mice (4 mon post-IRR), it is possible irradiated mice have consequently decreased forgetting (greater memory persistence) and also experience more proactive interference from past memories and would have less cognitive flexibility. Rodent cognitive flexibility can be directly tested using a reversal learning paradigm similar to the PD reversal learning task presented here. However, this task does not test rodent memory retention, and as we have shown, this relatively simplistic reversal learning is not affected by HZE radiation exposure. If the PD memory load were to be increased - for example, by training with more pairs of images - the rodent’s ability to then perform reversal with this larger number of stimuli would provide a more robust interrogation of cognitive flexibility. Alternatively, future experiments can hone in on dentate gyrus-specific cognitive flexibility via assessed LD reversal^39,42,58^, which contrasts with the PD reversal reliance on non-dentate gyrus brain regions (primarily PFC, perirhinal cortex, striatal circuits). Specifically, a challenging LD within-session reversal test would provide clarity as to whether IRR mice have decreased dentate gyrus specific-cognitive flexibility relative to controls^42^. Finally, future experiments could probe the influence of HZE particle exposure on the converse of pattern separation: pattern completion (i.e. formation of an accurate generalization of partial sensory input). Pattern separation and pattern completion abilities have a reciprocal relationship in both mice and aged humans^88,96^. As we show HZE particle exposure improves pattern separation (fine detail discrimination) and may increase proactive interference (given the decreased neurogenesis), it is possible irradiated mice have improved pattern separation yet worse pattern completion ability. If that were true, we could then further explore the possibility that the functional switch from pattern completion to pattern separation is driven in part by a slowing of the development of adult-generated neurons^88,97^. However, pattern completion relies on memory recall^88^, which is assessed in our PAL paradigm^41^ and is normal in our irradiated mice.

In conclusion, it is understandable that HZE particle exposure is presumed to have a negative influence on some lower and high-level cognitive functions, as many studies support this conclusion^12,14,33,40,98,99^. However, our study shows this is not universally true. Mature male mice that receive whole-body exposure to two different HZE particles perform similarly to control mice on many high-level cognitive tasks, reflecting the functional integrity of key neural circuits (i.e. PFC-perirhinal cortex-striatum, dorsal striatum, posterior cingulate cortex, hippocampus). Strikingly, mice irradiated with either ^56^Fe or ^28^Si actually perform “better” than control mice in both appetitive and aversive pattern separation tasks. Whether this HZE exposure-induced dentate gyrus-selective functional enhancement is compensation to earlier irradiation-induced neuromorphological changes^100^ remains to be tested, as does the task-, dose-, particle-, and LET-dependence of this functional enhancement. However, our work urges revisitation of the generally-accepted conclusion that space radiation is detrimental to cognition.

## Methods

### Animals

Animal procedures and husbandry were in accordance with the National Institutes of Health Guide for the Care and Use of Laboratory Animals, and performed in IACUC-approved facilities at UT Southwestern Medical Center (Dallas, TX), Children’s Hospital of Philadelphia (Philadelphia, PA), and Brookhaven National Laboratories (BNL, Upton NY). 2-month(mon)-old male C57BL/6J mice (JAX stock #000664) were housed at UTSW and shipped to BNL for irradiation at 6 mon of age. At both facilities, food and water were provided *ad libitum* except during the appetitive behavior tasks (see also Supporting Information [SI] text).

### Particle irradiation (IRR)

Mice received whole body HZE (^56^Fe or ^28^Si) particle IRR at BNL’s NASA Space Radiation Laboratory (NSRL). All mice were placed for 15 minutes (min) in modified clear polystyrene cubes (SI text). For ^56^Fe experiments, mice received Sham IRR (placed in cubes Monday, Wednesday, Friday, but received no IRR) or Fractionated (Frac) 20 cGy ^56^Fe (600 MeV/n, LET 174 KeV/μ, dose rate 20 cGy/min; placed in cubes and received 6.7 cGy on Monday, Wednesday, and Friday), and some experiments (Fig. 1) also included a group that received Non-Fractionated (Non-Frac) 20 cGy ^56^Fe (placed in cubes Monday, Wednesday, and Friday but received 20 cGy only on Friday). For ^28^Si IRR, mice received Sham IRR (placed in cubes, but received no IRR) or a single exposure of either 20 cGy or 100 cGy ^28^Si (275 MeV/n, LET 72 KeV/μ, dose rate 20 cGy/min or 100 cGy/min).

### Overview of behavioral testing

All mice began behavior testing 1-2-mon post-IRR, but within each cohort (Fig. 1a,e), the interval between radiation exposure and behavioral testing was equal for Sham and IRR groups. Parallel groups of mice were tested for appetitive touchscreen behavioral tests (operant touchscreen platform: touchscreen training; Pairwise Discrimination, PD; PD reversal; Location Discrimination, LD; different paired associates learning, PAL; Visuomotor Conditional Learning, VMCL) vs. aversive behavioral tests (contextual fear conditioning, CFC; contextual discrimination fear conditioning, CDFC). Subsets of mice were also tested for general activity (locomotor, LM), anxiety (dark/light box test, D/L) and pain sensitivity (pain threshold, PT; SI text).

#### Appetitive behavior testing

The touchscreen platform used was Model 80614 made by Lafayette Instruments (Lafayette, IN). Additional touchscreen methods are in SI text.

#### Aversive behavior testing

CDFC overview is provided below. See Figs. S2,S3 and SI text for additional CDFC information, and for detailed information about CFC.

#### Contextual discrimination fear conditioning (CDFC)

A modified CDFC behavioral paradigm was utilized in which mice were exposed daily to two contexts (Context A and B) that shared similarities (including a floor pattern, a high-salience contextual feature^55,88,101^ (SI text). Importantly, Context A was always paired with a foot shock, while Context B was never paired with a foot shock, as described below. Mice were exposed daily to both Context A and Context B for 16 days. The order of exposure to Context A and B alternated between days (Fig. S2).

### Tissue collection

After completion of behavioral tests, mice underwent intracardial perfusion, fixation, and tissue sectioning as previously described^47,102^ with additional detail provided in SI text.

### Immunohistochemistry (IHC)

Immunohistochemistry was performed as previously described^47,51^ with additional detail provided in SI text.

### Stereological cell quantification

Unbiased analysis of DCX + cell number was performed via stereologic quantification on a BX51 System Microscope (Olympus America, Center Valley, PA, USA) as previously described^47,51^.

### Statistical analyses

Data are reported as mean ± s.e.m. Testing of data assumptions (for example, normal distribution, similar variation between control and experimental groups, etc.) and statistical analyses were performed in GraphPad Prism (ver. 8.2.0). Statistical approaches and results are provided in Table S1 for main figures and in Table S2 for supplementary figures, and statistical analysis summaries are provided in the figure legends. Analyses with two groups were performed using an unpaired, two-tailed Student’s t-test and analyses with more than two groups and one variable were performed using one-way ANOVA and Bonferroni post hoc test. Analyses with more than two variables were performed using two-way ANOVA with Bonferroni post hoc test; repeated measures (RM) were used where appropriate, as indicated in figure legends and Tables S1, S2. For the distribution of subjects reaching criteria between control and experimental groups, the Mantel-Cox test was used, and significance was defined as *p < 0.05. For behavioral studies, mice were randomly assigned to groups. Sample sizes were pre-determined via power analysis and confirmed on the basis of extensive laboratory experience and consultation with CHOP and PennMed statisticians.

### Datasets

Raw data are made available to researchers on written request.

### Ethics

#### Human subjects

No

#### Animal subjects

Yes

### Ethics statement

The study was approved by three Ethics committees (the Institutional Animal Care and Use Committees at the University of Texas Southwestern Medical Center [UTSW], Children’s Hospital of Philadelphia [CHOP], and Brookhaven National Laboratories [BNL]). Specifically, animal procedures and husbandry were in accordance with the National Institutes of Health Guide for the Care and Use of Laboratory Animals, and performed in IACUC-approved facilities at UT Southwestern Medical Center (UTSW, Dallas TX; AAALAC Accreditation #000673, PHS Animal Welfare Assurance D16–00296, Office of Laboratory Animal Welfare [OLAW] A3472-01), Children’s Hospital of Philadelphia (CHOP, Philadelphia, PA; AAALAC Accreditation #000427, PHS Animal Welfare Assurance D16-00280 [OLAW A3442-01]) and Brookhaven National Laboratories (BNL, Upton NY; AAALAC Accreditation #000048, PHS Animal Welfare Assurance D16-00067 [OLAW A3106-01]).

### Dual-use research

Not applicable.

### Permissions

This manuscript represents original work, and is not a reproduction or modification of any part of an article that has been previously published or submitted to another journal.

## Supplementary information

Supplementary Information.
