# Supplementary material for: Multi-domain cognitive assessment of male mice shows space radiation is not harmful to high-level cognition and actually improves pattern separation
Source: Sci Rep. 2020 Feb 17;10:2737. doi: 10.1038/s41598-020-59419-z (PMC7026431; doi:10.1038/s41598-020-59419-z)
Supplement: Supplementary file 1 — Supplementary Information. [file 41598_2020_59419_MOESM1_ESM.pdf]

SUPPORTING INFORMATION APPENDIX for

Multi-domain cognitive assessment of male mice shows space radiation is not harmful to high-level cognition and actually improves pattern separation

Cody W. Whoolery[+], Sanghee Yun[+], Ryan P. Reynolds, Melanie J. Lucero, Ivan Soler, Fionya H. Tran, Naoki Ito, Rachel L. Redfield, Devon R. Richardson, Hung-ying Shih, Phillip D. Rivera, Benjamin P. C. Chen, Shari G. Birnbaum, Ann M. Stowe, Amelia J. Eisch

[+]*These authors contributed equally to this work*

Contents:

Supporting Information
Figures S1-S3 (legends provided on same page as Supporting Figures)
Tables S1-S2

SUPPORTING INFORMATION

Animals

Animal procedures and husbandry were in accordance with the National Institutes of Health Guide for the Care and Use of Laboratory Animals, and performed in IACUC-approved facilities at UT Southwestern Medical Center (UTSW, Dallas TX; AAALAC Accreditation #000673, PHS Animal Welfare Assurance D16-00296, Office of Laboratory Animal Welfare [OLAW] A3472-01), Children's Hospital of Philadelphia (CHOP, Philadelphia, PA; AAALAC Accreditation #000427, PHS Animal Welfare Assurance D16-00280 [OLAW A3442-01]) and Brookhaven National Laboratories (BNL, Upton NY; AAALAC Accreditation #000048, PHS Animal Welfare Assurance D16-00067 [OLAW A3106-01]). 2-month(mon)-old male C57BL/6J mice (Jackson Laboratories, stock #000664) were housed at UTSW and shipped to BNL for irradiation at 6 mon of age. During shipping and housing at BNL, mice were provided Shepherd Shacks (Bio-Serv). Mice were housed at UTSW or BNL (3-4/cage, light on 06:00, lights off 18:00, UTSW: room temperature 68-79°F, room humidity 30-70%, BNL: room temperature 70-74°F and room humidity 30-70%). At both facilities, food and water were provided *ad libitum* except during the appetitive behavior tasks.

Particle irradiation (IRR)

Mice received whole body HZE particle irradiation (IRR) at BNL's NASA Space Radiation Laboratory (NSRL) during NSRL campaigns 12C, 13A, 13B, 16B, and 18A. The ^{56}Fe and ^{28}Si ion beams were produced by the AGS Booster Accelerator at BNL and transferred to the experimental beam line in the NSRL. Delivered doses were ±0.5% of the requested value. All mice - regardless of whether control (Sham) or experimental - were placed for 15 minutes (min) in modified clear polystyrene rectangular containers (AMAC Plastics, Cat #100C, W 5.8 x L 5.8 x H 10.6 cm; modified with ten 5-mm air holes). For ^{56}Fe experiments, mice received Sham IRR (placed in cubes Monday, Wednesday, Friday, but received no IRR) or Fractionated (Frac) 20 cGy ^{56}Fe (600 MeV/n, LET 174 KeV/μ, dose rate 20 cGy/min; placed in cubes and received 6.7 cGy on Monday, Wednesday, and Friday), with some experiments including a group that received Non-Fractionated (Non-Frac) 20 cGy ^{56}Fe (placed in rectangular containers Monday, Wednesday, and Friday but received 20 cGy only on Friday). For ^{28}Si IRR, mice received Sham IRR (placed in rectangular containers, but received no IRR) or a single exposure of either 20 cGy or 100 cGy ^{28}Si (275 MeV/n, LET 72 KeV/μ, dose rate 20 cGy/min or 100 cGy/min). Post-IRR, mice were returned to UTSW and housed in quarantine for 1-2 mon prior to initiation of behavior testing. Body weights **(Fig. S1a)** were taken multiple times: prior to irradiation, at irradiation, and at least monthly post-IRR until collection of brain tissue.

Overview of behavioral testing
All mice began behavior testing 1-2-mon post-IRR. Parallel groups of mice were tested for appetitive touchscreen behavioral tests (operant touchscreen platform: touchscreen training; Pairwise Discrimination, PD; PD reversal; Location Discrimination, LD; different paired associates learning, PAL; Visuomotor Conditional Learning, VMCL) vs. aversive behavioral tests (contextual fear conditioning, CFC; contextual discrimination fear conditioning, CDFC). Subsets of mice were also tested for general activity (locomotor, LM), anxiety (dark/light box test, D/L) and pain sensitivity (pain threshold, PT), methods for which are provided below.

Appetitive Behavior Testing
Touchscreen behavior tests (Abet II software, Cat. #89505).
Touchscreen platform and software. Software used for the touchscreen system was ABET II (Lafayette Instruments, Cat. #89505), and individual ABET programs for specific touchscreen training and testing sessions are below**.** Sham and IRR mice were trained on an operant touchscreen platform (TS training). The touchscreen platform used was Model 80614 made by Lafayette Instruments (Lafayette, IN). Each operant chamber is encased in a sound-attenuating chamber. Each chamber is trapezoid-shaped, with the widest wall serving as the "touchscreen" (W 238 x H 170 mm) and the opposite and narrowest wall (W 46 mm) containing a motion-sensitive center dispenser (tray) to deliver liquid reward (Strawberry Ensure, Abbott Laboratories, Chicago, IL). Each chamber has two lights (tray light and overhead house light), and is equipped with a speaker (ceiling in each chamber) to play a tone. Aside from initial priming reward used during training, a "reward" is defined as 7-ul Ensure delivered to the illuminated tray at the same time as a tone is played. Aside from training sessions, the term "initiate a trial" is defined as the mouse placing its head in the tray when the tray light is illuminated and the tone is played. The two remaining walls of the chamber are infrared-permeable to track rodents during testing. The floor is a perforated metal grid, and the solid roof is hinged for easy placement/removal of the animal. A computer outside of the chamber controls the programs and recording of each session. Mice are tested in their light cycle Monday through Friday until testing was complete. Software used for the Touchscreen System is from ABET II (Lafayette Instruments, Cat. #89505), and individual ABET programs for specific touchscreen training and testing sessions are listed below.

Food exposure/restriction. TS training and testing occurred Monday through Friday during the light cycle. Mice were maintained on a food-restricted diet. In brief, mouse chow (16% protein 2016 Teklad Global Diet, Envigo, Madison, WI) was removed from each cage at 5 pm the day prior to training or testing. Each cage was given *ad libitum* access to chow for 3 hour (hr; minimum) to 4 hr (maximum) immediately following daily touchscreen training/testing, and from completion of training/testing on Friday until Sunday 5 pm. Mice were weighed each Wednesday to ensure weights >80% initial body weight. While weights below this threshold merited removal of the mouse from the study, zero mice reached this threshold (**Fig. S1a**).

Touchscreen training (**Fig. 2a, 3a**) consists of 5 steps, as previously published[1–3]: Habituation (Hab), Initial Touch (IT), Must Touch (MT), Must Initiate (MI), and Punish Incorrect (PI). Methods for each step of the touchscreen training are described in turn below.

Habituation (Hab). Mice are placed in touchscreen chamber for 30-min (max) session with the tray light turned on (LED Light, 75.2 lux). For the initial reward in each habituation session, a tone is played (70 decibel [dB] at 500 Hz, 1000 ms) at the same time as a priming reward (150-ul Ensure) is dispensed to the chamber tray. After the mouse inserted its head and removed its head from tray, the tray light turns off and a 10-s delay begins. At the end of the delay, the tray light is turned on and tone is played again as a standard reward (7-ul Ensure) is dispensed. If the mouse's head remains in the

tray at the end of the 10-s delay, an additional 1-s delay is added. Mice complete Habituation training after they collect 25 rewards (25 x 7 ul) within 30 min. Mice that achieve habituation criteria faster than 30 min are removed from the chamber immediately after their 25th reward in order to minimize extinction learning.

Initial Touch (IT). Drawing from a bank of 40 preselected black and white images (240 x 240 pixels), a random image is displayed on the screen in a pseudo-random location such that no image is displayed in that location more than 3 consecutive times. The mouse has 30 s to touch the image (typically with their nose). If the mouse does not touch the image, the image is removed, a reward (7ul Ensure) is delivered into a lit tray, and a tone is played. After the reward is collected, the tray light turns off and a 20-s intertrial interval begins. If the mouse touches the image on the screen while it is displayed, the image is removed and the mouse receives 3 times the normal reward (21-ul Ensure, tray lit, tone played). Mice advance from Initial Touch training after they complete 25 trials (irrespective of reward level received) within 30 min. Mice that achieve Initial Touch criteria faster than 30 min are removed from the chamber immediately after their 25th trial.

Must Touch (MT). Similar to Initial Touch training, a random image is displayed, but now the image remains on the screen until it is touched. If the mouse touches the screen, the mouse receives a reward (7-ul Ensure, tray lit, tone played). If the mouse touches the blank screen, there is no response (no reward dispensed, no light in tray, no tone). Mice advance from Must Touch training after they complete 25 trials within 30 min. Mice that achieve Must Touch criteria faster than 30 min are removed from the chamber immediately after their 25th trial.

Must Initiate (MI). Must Initiate training is similar to Must Touch training, but a mouse is now required to initiate the training by placing its head into the already-lit tray. A random image from the image bank will then appear on the screen, and the mouse must touch the image to receive a reward (7-ul Ensure, tray lit, tone played). Following the collection of the reward, the mouse must remove its head from the tray and then reinsert its head to initiate the next trial. Mice advance from Must Initiate training after they complete 25 trials within 30 min. Mice that achieve Must Initiate criteria faster than 30 min are removed from the chamber immediately after their 25th trial.

Punish Incorrect (PI). Punish Incorrect training builds on Must Initiate training, but here if a mouse touches a portion of the screen that is blank (does not have an image), the overhead house light turns on and the image disappears from the screen. After a 5-s timeout period, the house light turns off, and the mouse has to initiate a correction trial, where the same image appears in the same location on the screen. The correction trials are repeated until mouse successfully presses the image but are not counted towards the final percent correct criteria. Mice advance from Punish Incorrect training and onto testing after they complete 25 trials within 30 min at ≥76% (≥19 correct) for two consecutive days. Mice that achieve Punish Incorrect criteria faster than 30 min are removed from the chamber immediately after their 25th trial.

Pairwise Discrimination (PD)/Reversal Testing (ABET II software, Cat. #89540). After training on the touchscreen platform, mice went through PD/PD Reversal tests **(Fig. 2)**. For PD, two images from the image bank that the mice had never seen before were simultaneously presented on the screen (i.e. plane vs. spider). Only one of these stimulus images was rewarded (S+), and the image that was rewarded was counterbalanced within each group of mice. After the mouse initiated the trial, the rewarded image was presented on either the left or right side of the screen. The presentation side was pseudo-randomly selected such that the S+ was not presented on the same side more than 3 times in a row. An incorrect choice led to a correction trial, and the mouse had to repeat the trial until it correctly selected the rewarded image displayed in the same location. The correction trial was not counted towards the final percent of trials correct. For Reversal testing, the S+ and S- were switched so that the previously-rewarded S+ stimulus image was now no longer rewarded. The mouse performed PD or Reversal testing until it was able to complete 24 trials in 30 min at >76% accuracy

(Day 1) and >80% (Day 2) for 2 days in a row. For PD and PD reversal data, Days 1 and 6 and the last day or Day 1, 8, 12, and last day were reported, respectively. Day to completion indicated average of days to reach criteria. Distribution of proportion of subjects which reach criteria was plotted over all testing days. Session length (presented in s), percent (%) correct, the number of errors (number of correction trials) are also reported.

Paired associates learning (PAL, ABET II software, Cat. #89541). Once mice achieved all five stages of general touchscreen training using the three-window mask **(Fig. 1b, 3a)**, they began training in and assessment on object-location different paired-associates learning (PAL). There were three possible stimulus ‘objects’ (images of a flower, plane, or spider) and three possible positions on the screen (left, middle, or right) **(Fig. 3b)**. All ‘objects’ had a correct ‘location’ that was unique to them. Two stimuli were displayed at the same time during a trial. One was in the correct location (S+) and the other was in the incorrect location (S-), and whether a stimulus was correct was determined by the location in which it was presented (e.g. flower/left; plane/middle; spider/right). If the mouse nose-poked the incorrect stimulus, no reward was delivered and a 5-s time-out followed before the mouse was given the opportunity to complete a correction trial. Correction trials continued until the correct stimulus was chosen. A correction trial (the number of errors) consisted of representation of the stimulus array in the same location configuration. Correction trials were not included in the percent correct. Each session was complete when the mouse performed 25 trials or 30 min had elapsed. PAL lasted for 29 days, and measures reported include session length, completed trial number, percent correct, and number of errors.

Visuomotor Conditional Learning (VMCL, ABET software, Cat #89542). For VMCL, mice received one additional training step, termed VMCL train, prior to the VMCL test.

VMCL train. VMCL train is designed to teach the mouse to touch two images on the screen in a specific order and in rapid succession. The first touch must be to an image presented in the center of the screen, and the second touch must be to an image presented either on the left or right of the screen. Specifically, after trial initiation, the mouse must touch a center white square (200 x 200 pixels), which then disappears after touch. A second white square immediately appears on either the left or right side of the screen in a pseudorandom style, such that a square is located on each side 5 out of 10 times, but not more than 3 times in a row. If the mouse selects the location with the second white square, a reward is provided, and a 20-s inter-trial interval starts. However, if the mouse selects the location without a square, then the second stimulus is removed, and the house light illuminates for 5 s to indicate a timeout period which must conclude prior to the 20-s inter-trial interval. Then the mouse is presented with a correction trial which must be completed prior to a new set of locations being displayed. VMCL train is complete when the mouse completes 2 consecutive days of 25 trials in 30 min with >75% correct. Session length (presented in s), trial number, percent correct, and number of errors are reported on the first and the last day of VMCL train. Day to completion indicates average of days to reach criteria. Distribution of proportion of subjects which reach criteria is plotted over entire train days.

VMCL test. Mice are provided with a center black-and-white image (spikes or horizontal bars, **Fig. 3g, h**). Once touched, the center image disappears and white squares appear on both the right and left of the screen. For this task, the center image of the spikes signals that the rodent should touch the right square, while the center image of the horizontal bars signals that the rodents should touch the left square. The two center images are presented pseudorandomly for an equal number of times, and the mice have 2 s to touch the white square on the right or left side of the central image. If they fail to touch the white square within 2 s, a timeout period begins. The same timeout and inter-trial-intervals are used for VMCL testing as were used for VMCL train. As with VMCL train, VMCL test correction trials are used to protect against side bias. VMCL testing is complete when the

mouse completes 2 consecutive days of 25 trials in 30 min with ≥76% correct (Day 1) and ≥80% correct (Day 2) in a row. Session length (presented in s), trial number, percent correct, percent missed, and number of errors are reported for Day 1 and 8 and the last day of VMCL test. Day to completion indicates average of days to reach criteria. Distribution of proportion of subjects which reach criteria is plotted over all VMCL test days.

Location Discrimination (LD; ABET2 software, Cat #89546-6). For LD, mice receive one additional training step, termed LD1-choice, prior to the actual 2-choice LD test (LD2).

LD train. Mice initiate the trial, which leads to the display of two identical white squares (25 x 25 pixels, **Fig. 4a**) presented with two black squares between them, a separation which is termed “intermediate” in difficulty (8th and 11th windows in 6 X 2 high grid-bottom row). One of the locations of the squares is rewarded (L+) and the other is not, and the L+ location (left or right) is counterbalanced within-group. On subsequent days, the rewarded square location is switched (becomes L-), then L+, then L-, etc. A daily LD train session is complete once the mouse touches either L+ or L- 25 times or when 30 min has passed. Once the animal reaches 25 trials in 30 min for 2 consecutive days (irrespective of accuracy), the mouse advanced to the LD 2-choice random test. Session length and percent correct on the last day of LD train are reported. Days to completion indicates average of days to reach criteria and distribution of proportion of subjects which reach criteria is plotted over entire training days.

LD test. Mice initiate the trial, which leads to the display of two identical white squares, either with four black squares between them (“large” separation, two at maximum separation (7th and 12th windows in 6 x 2 high grid-bottom row) or directly next to each other (“small” separation, two at minimum separation (9th and 10th windows in the Bussey Mouse Operant Mode 6 x 2 high grid-bottom row; **Fig. 4f**). Like the LD 1 train, only one of the square locations (right-most or left-most) is rewarded (L+, same side for both large and small separations, and counterbalanced within-groups). The rewarded square location is switched the following day, and the location continues to alternate daily throughout testing. Each day, the separation (large vs. small) is pseudorandomly displayed (same separation shown no more than 3 consecutive times). LD testing is complete when the mouse completes 45 trials in 30 min regardless of accuracy. Session metrics reported are length, percent correct, number of completed trials, number of blank touches, reward collection latency (time between reward presentation and the first head entry into the reward port), and correct/incorrect image response latency (latency from correct/incorrect image response). For analysis of performance in 10-trial Blocks (1st 10-trial Block: 1-10 trials, 2nd 10-trial Block: 11-20 trials, 3rd 10-trial Block: 21-30 trials, 4th 10-trial Block: 31-40 trials) on the last day, metrics reported are duration, percent correct, blank touch, and left and right touch during inter-trial-interval. Days to completion indicates average of days to reach criteria and distribution of proportion of subjects which reach criteria is plotted over all LD test days.

Aversive Behavior Testing

Contextual Discrimination Fear Conditioning (CDFC). CDFC paradigm and chambers are shown and described in **Figure S2**. “Context A” consisted of a standard fear conditioning chamber (Med Associates) outfitted with a grid floor and white overhead house light, was scented with vanilla, and was paired with a shock. “Context B” consisted of a standard fear conditioning chamber with a grid floor, but with a near-infrared light and a black A-frame insert, was scented with mint, and was not paired with a shock. There were other subtle differences between the contexts. For example, prior to placement into Context A, mice were individually placed into a transfer cage (a standard cage with bedding), and then placed by the tail into Chamber A. After exposure to Context A, the mouse was removed and Context A was cleaned with Coverage Plus NPD solution (Steris, Mentor, OH). In contrast, prior to placement into Context B, mice were individually placed into a transfer cage lined

with white paper towels, and each mouse was scooped by hand into both the transfer cage and testing chamber. After exposure to Context B, the mouse was removed and Context B was cleaned with 1% acetic acid. Each twice daily exposure over 16 days lasted 4 min 2 s, during which freezing behavior was scored for the first 3 min. Mice in Context A, but not Context B, received a single, mild foot shock (0.25 mA, 2-s duration) after 3 min in the context. Mice then remained in the chambers for one additional minute until the session was complete. The interval between daily exposures to Context A or B was 2-2.5 hrs. During the test, mice were exposed daily to both Context A and Context B. The order of exposure to Context A and B alternated between days (BAABABBABAABABBA) such that on days 2, 3, 5, 8, 10, 11, 13, and 16 mice were exposed to Context A first and Context B second **(Fig. S2)**. For CDFC data analysis, the percent freezing in Context A and Context B were measured each day, and data from each treatment group were collapsed and averaged across every two days, referred to as Blocks. Therefore, data were analyzed as 8 Blocks (16 testing days) such that the grouping of days into Blocks was as follows: [BA AB] [AB BA] [BA AB] [AB BA] etc. However, since Day 1 of exposure includes data from mice prior to their first tone/shock pairing and therefore their response does not reflect a learned association, Block 1 (Days 1-2) was removed from analysis. Percent of time freezing was measured using linear analysis. The threshold for freezing was 20 arbitrary units detected using the proprietary Med Associates Software. Additional analysis parameters include bout length (0.5 s) and frames/s (30).

<u>Contextual Fear Conditioning (CFC)</u>. CFC paradigm and chambers are shown and described in **Figure S3**. CFC consisted of two phases: training (Day 1), and testing (Day 2-3). Mice were habituated to the behavior room environment 1 hr each day prior to training and testing sessions. On Day 1, mice were trained to associate a novel context (standard fear conditioning chamber, grid flooring, no odor, house lights on; Med Associates Inc., St. Albans, VT) with a shock. Two minutes after placement in the novel context, an auditory cue was played (80-dB white noise, 30-s duration, Med Associates Inc.), which co-terminated with the presentation of a 0.5-mA shock (2-s duration). This cue-shock pairing was repeated twice during Day 1 (5-min training session), with 1 min between the cue-shock presentations. On Day 2, mice underwent context testing: 5 min in the same environment as Day 1 training, but no auditory cue or foot shock presented. On Day 3 (^{56}Fe IRR mice) mice underwent auditory cue testing: 6 min in another novel context (plastic flooring, triangular roof, vanilla odor, house lights on). For training and testing sessions, freezing behavior was assessed using VideoFreeze software (Med Associates Inc.), compiled for each phase of each session (e.g. Pre-Cue, During Cue, etc.), and presented as percent percent time freezing for each phase.

General Behavioral Tests
<u>Locomotor Activity (LM)</u>. Within 2-mon post-IRR (^{56}Fe experiments: 59-days post-IRR; ^{28}Si experiments: 49-days post-IRR), mice underwent a single locomotor activity recording session from 5pm-9am under red light. After 1-hr acclimation to the testing suite, group-housed mice were individually placed into clean standard cages and were given *ad libitum* food and water. Beam breaks were recorded over 16 hr using the Photobeam Activity System-Home Cage (San Diego Instruments; San Diego, CA). Data were collapsed into 30-min bins across the 16-hr session, and are presented as number of beam breaks. At the completion of recording, mice were placed back to their original group-housed cage and returned to their normal housing room.

<u>Dark/Light test (D/L)</u>. The apparatus consisted of a polypropylene cage (L 44 x W 21 x H 21 cm) unequally divided (⅔ and ⅓) into two chambers. The large chamber was white and brightly-illuminated by two 20-W fluorescent lights (1388 lux at cage floor), while the small chamber was dark (not illuminated). Initially the mouse was placed in the dark side for 2 min, after which the door between the two chambers is opened and the transitions of the mouse between the two

chambers and time in each chamber was detected for 10 min by seven photocells. The time spent in the brightly-lit side and latency to enter the brightly-lit side were measured by an automated system (Med Associates).

Pain Threshold (PT). Mice were individually placed into boxes equipped with a metal grid floor connected to a scrambled shock generator (Med Associates Inc., St. Albans, VT). After ~1 min, mice received a series of foot shocks (each 2-s duration) with increasing intensity. The initial shock intensity was 0.05 mA, and the amplitude was increased by 0.05 mA for each consecutive foot shock with a 15-s intershock interval. The first shock intensity at which each animal displayed each behavior (flinch, vocalization, or jump) is reported. Once the animal displayed all three behaviors, it was removed from the chamber.

Tissue Collection

After completion of behavioral tests, mice underwent intracardial perfusion and fixation as previously described[4,5]. ^{56}Fe IRR mice were perfused 4-6-mon post-IRR (10 to 12 mon of age) and ^{28}Si IRR mice were perfused 6-mon post-IRR (14 mon of age). Briefly, mice were anesthetized with chloral hydrate (Sigma-Aldrich cat. #C8383, 400 mg/kg, stock solution 400 mg/ml made in 0.9 % NaCl solution, i.p.) and exsanguinated intracardially with 0.1M PBS (7 ml/min, 6 min) and followed by perfusion intracardially with 4% paraformaldehyde in 0.1M PBS (7 ml/min, 15 min). As stress can influence neurogenesis and thus doublecortin-immunoreactive (DCX+) cell number, steps were taken to minimize potential stress differences among mice in the same cage: each cage was gently removed from the housing room and brought to the adjacent procedure room immediately prior to anesthesia; mouse cage transfer was performed by a researcher with clean personal protective equipment; and all mice in a cage were anesthetized within 3 min and began exsanguination within 5 min of being brought into the procedure room. With these and other steps, we have found neurogenesis levels in mice can be reliably and accurately evaluated. Brains were harvested and placed in 4% paraformaldehyde at room temperature for 2 days, transferred to cryoprotectant (30% sucrose in 0.1 M PBS and 0.1% NaN_3) and stored at 4°C until sectioning. Brains were coronally sectioned on a freezing microtome (Leica), with 30 μm sections collected in serial sets through the entire anterior-posterior length of the hippocampus (distance range from Bregma: -0.82 to -4.24 μm)[6]. These eight serial sets of sections (section sampling fraction, ⅛) were stored in 0.1% NaN3 in 1x PBS (Fisher Scientific; Pittsburgh, PA) at 4°C until processed.

Immunohistochemistry (IHC). One complete set of coronal sections from a 1:n series (1:8 or 1:9) was mounted onto glass slides (Superfrost/Plus, Fisher) in rostral to caudal order and allowed to dry. To visualize DCX+ cells using 3’3-diaminobenzidine (DAB), slide-mounted sections were treated for antigen retrieval (0.01M citric acid in MQH_2O, pH 6.0, 95°C, 15 min) and quenching of endogenous peroxidases (0.3% hydrogen peroxide in 1xPBS, 30 min). Non-specific staining was blocked by incubation in 3% normal donkey serum (NDS) and 0.1% Triton X-100 in 1xPBS for 60 min. Sections were then incubated in goat-anti-DCX primary antibody (1:500, Santa Cruz) overnight at room temperature in 3% NDS, 0.1% Tween-20 in 1xPBS. The following day, sections were incubated for 60 min with biotinylated donkey anti-goat antibody (1:200, Jackson ImmunoResearch) in 1.5% normal donkey serum in 1xPBS followed by rinses. A 60-min incubation in avidin-biotin complex (ABC Elite, 1:50, Vector Laboratories) was then performed, followed by visualization of immunoreactive cells using DAB (Thermo Scientific Pierce) and Nuclear Fast Red counterstaining (Vector Laboratories). Tissue was then dehydrated with a series of increasing ethanol concentrations and defatted section (Citrosolv) were cover slipped with DPX Mountant (Sigma-Aldrich).

Figure Preparation

For graphical data, figures for each data set were produced in Prism (GraphPad ver. 8.2.0) and transferred to Illustrator (Adobe Illustrator cc2018 version 22.1) to enable uniform line thickness and figure size. For photomicrographs, immunostained sections were visualized with an epifluorescence microscope (Olympus BX51) with 10x and 40x objectives and images were captured with the Olympus DP Manager Program before being prepared in Adobe Illustrator 2018 (version 22.1).

Transparency and Reproducibility

Behavioral experiments were performed by researchers blind to treatment (Sham or IRR), which was feasible since such the low doses of space radiation used here do not have gross measurable impact on mouse weight or hair loss. Automated scoring was used for most behavior tests. Touchscreen testing criteria was based on rodent performance, thus avoiding scoring discrepancies among researchers. For immunohistochemical experiments, tissue was coded to obscure treatment information, and codes were not broken until data analyses were complete. After publication, raw data and images will be made available to interested researchers.

References for Supporting Information

1. Horner, A. E. *et al.* The touchscreen operant platform for testing learning and memory in rats and mice. *Nat. Protoc.* **8**, 1961–1984 (2013).
2. Mar, A. C. *et al.* The touchscreen operant platform for assessing executive function in rats and mice. *Nat. Protoc.* **8**, 1985–2005 (2013).
3. Oomen, C. A. *et al.* The touchscreen operant platform for testing working memory and pattern separation in rats and mice. *Nat. Protoc.* **8**, 2006–2021 (2013).
4. Yun, S. *et al.* Stimulation of entorhinal cortex-dentate gyrus circuitry is antidepressive. *Nat. Med.* **24**, 658–666 (2018).
5. Lagace, D. C. *et al.* Adult hippocampal neurogenesis is functionally important for stress-induced social avoidance. *Proc. Natl. Acad. Sci. U. S. A.* **107**, 4436–4441 (2010).
6. Franklin, K. B. J., Paxinos, G. & Others. *The mouse brain in stereotaxic coordinates*. vol. 3 (Academic press New York:, 2008).

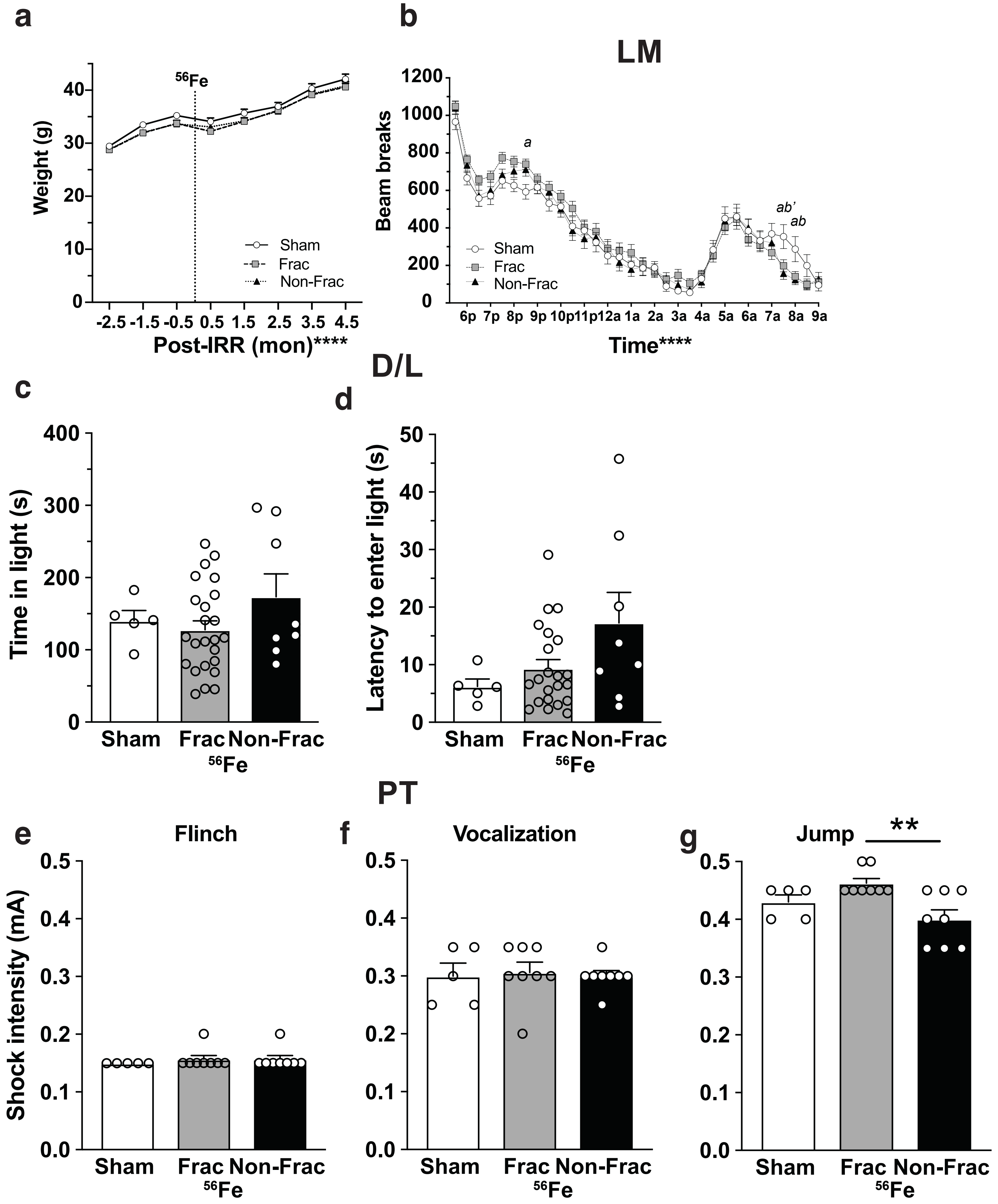

Figure S1. Weights, locomotion, anxiety, and pain threshold are generally unaffected in mice exposed to whole body Frac or Non-Frac ^{56}Fe radiation in maturity. (a) No gross weight difference was detected before and after radiation in Sham or ^{56}Fe groups. **(b-g)** Locomotor activity (LM) measured in 30 minute bins for 16 hrs **(b)**, time spent in light **(c)**, latency to enter light **(d)** in dark/light box (D/L) and measurements for flinch **(e)**, vocalize **(f)**, and jump **(g)** in the pain threshold test (PT) reveal no gross changes after exposure to Sham or ^{56}Fe radiation. Mean±SEM. Statistical analysis in **a, b**: Two-way RM measures ANOVA, **** $p<0.0001$, Bonferroni's post-hoc analysis, *a* $p<0.05$ in Sham vs Frac; *b* $p<0.05$, *b'* $p<0.01$ in Sham vs Non-Frac. One-way ANOVA in **c-g**, Bonferroni's post-hoc analysis. ** $p<0.01$. a=A.M., Frac=fractionated, months=mon, mA=milliampere, p=P.M., Non-Frac=non-fractionated, s=seconds.

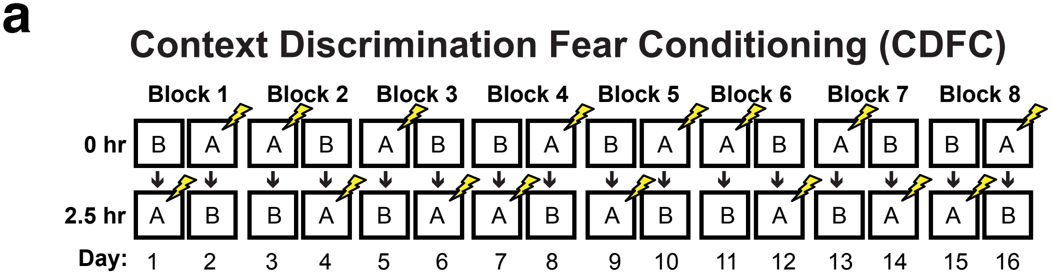

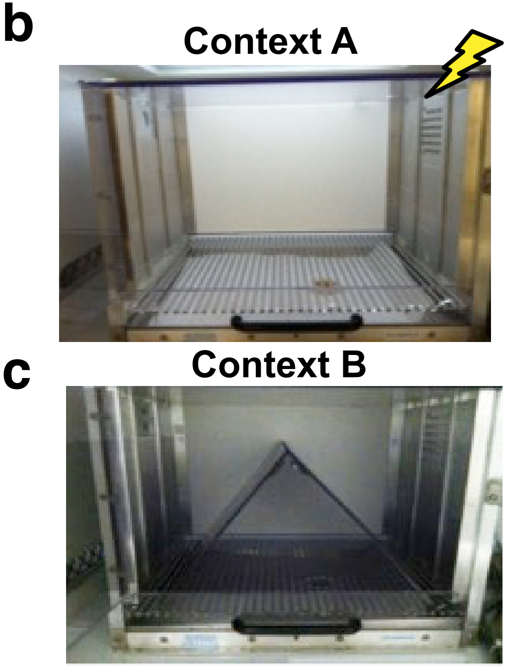

d

CDFC parameters	Context A	Context B
**Chamber characteristic**		
Floor	Grid bars	Grid bars
Wall & ceiling	Standard	Triangular
Lighting	White light	Infrared
Odor	Vanilla	Mint
Holding cage	Bedding	Paper towel
**Shock amplitude**	0.25 mA	-
**Number of shocks**	1	0
**Shock length**	2 sec	-
**Latency to shock**	3 min	-
**Length of paradigm**	4 min	4 min

Figure S2. Contextual Discrimination Fear Conditioning (CDFC) paradigm. (a) Sixteen-day CDFC paradigm depicting daily, randomized placement into Context A (shock-paired, indicated by yellow lightning bolt) and the contextually-similar Context B (no shock). **(b-c)** Photographs of chamber set up as Context A (**b**, the context paired with mild foot shock) and Context B (**c**, a somewhat distinct context that is never paired with a foot shock). **(d)** Table of parameters of Context A and Context B used for this CDFC paradigm. “-”=not applicable.

a

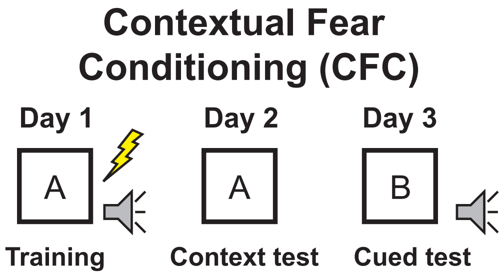

b

CFC parameters	Training	Context test	Cued test
**Chamber characteristic**			
Floor	Grid bars	Grid bars	Plastic floor
Wall & ceiling	Standard	Standard	Triangular
Lighting	White light	White light	White light
Odor	-	-	Vanilla
**Shock amplitude**	0.5 mA	-	-
**Number of shocks**	2	0	0
**Shock length**	2 sec	-	-
**Latency to first shock**	2.5 min	-	-
**Interval between shocks**	1.5 min	-	-
**Auditory sound**	30 sec white noise	-	3 min white noise
**Total test time**	5 min	5 min	6 min

c **d**

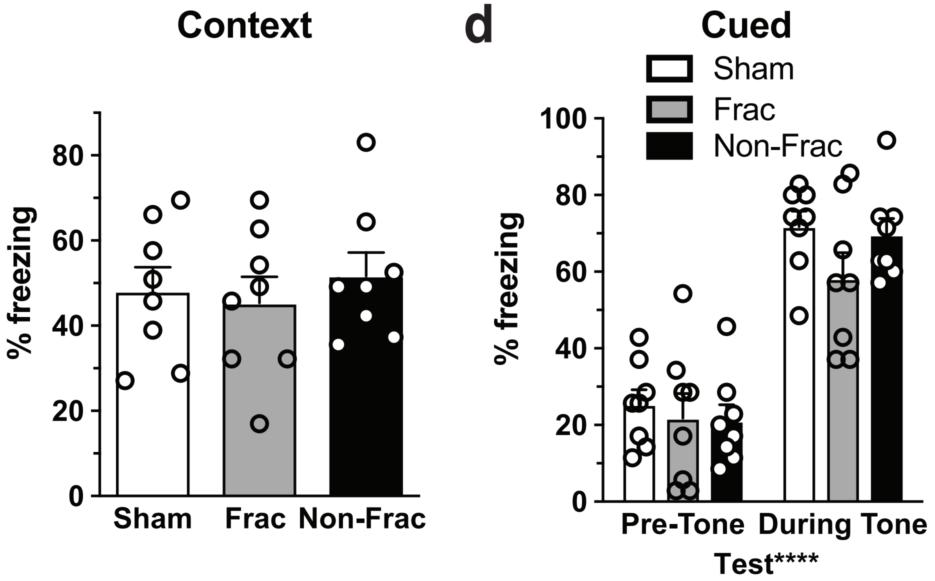

Figure S3. Contextual fear conditioning (CFC) is unaffected in mice exposed to whole body Fractionated (Frac) or Non-Fractionated (Non-Frac) 20 cGy ^{56}Fe radiation. **(a)** Three-day CFC paradigm depicting placement (Day 1) into in novel context which is paired with a cue (auditory tone, indicated by grey speaker, is paired with shock, indicated by yellow lightning bolt) followed by testing in the same context (Day 2) and in an additional novel context for cued testing (Day 3). **(b)** Table of parameters of the contexts used for training and testing in this CFC paradigm. **(c-d)** Percent freezing in response to context **(a)** or cue **(b)** in the CFC test reveals a lack of effect with ^{56}Fe radiation. Mean±SEM. **(c)** One-way ANOVA, **(d)** Two-way repeated measures ANOVA. ****p>0.0001, “-”=not applicable.

Table S1. Reporting statistical results of main figures

Subject	Figure	n	Mean	Statistics (variables)	Main Effect	F Value	P value	Post hoc Test
General Touchscreen Training w/two windows	2a	Sham: 12<br>Frac 20 cGy: 12	Training Stage: HAB, IT, MT, MI, PI<br>Sham: 1, 1, 2.25, 1.25, 6.25<br>Frac 20 cGy: 1, 1, 2.5, 3.083, 3.833	Two-way RM ANOVA	interaction<br>training stage<br>treatment<br>subject	F (4, 88) = 3.169<br>F (4, 88) = 14.98<br>F (1, 22) = 0.03139<br>F (22, 88) = 0.9710	P=0.0175<br>P<0.0001<br>P=0.861<br>P=0.5076	Bonferroni<br>*PI: P<0.05
Pairwise Discrimination (PD)/PD Reversal (Rev)	2d	Sham: 12<br>Frac 20 cGy: 12	PD, Rev<br>Sham: 9.083, 15.83<br>Frac 20 cGy: 10.92, 14.58	Two-way RM ANOVA	interaction<br>test type<br>treatment<br>subject	F (1, 22) = 2.642<br>F (1, 22) = 30.16<br>F (1, 22) = 0.03605<br>F (22, 22) = 2.623	P=0.1183<br>P<0.0001<br>P=0.8512<br>P=0.0141	Bonferroni
PD Reversal (Rev)	2e	Sham: 12<br>Frac 20 cGy: 12	Rev<br>Sham: Median: 9.5<br>Frac 20 cGy: Median: 12	Log-rank (Mantel-Cox) test	NA	NA	P=0.0433	NA
Pairwise Discrimination (PD)	2f	Sham: 12<br>Frac 20 cGy: 12	PD<br>Day of reversal: 1, 6, Last<br>Sham: 1800, 1657, 1396<br>Frac 20 cGy: 1800, 1744, 1351	Two-way ANOVA	interaction<br>day of testing<br>treatment	F (2, 41) = 43.97<br>F (1, 22) = 0.08425<br>F (2, 41) = 0.9220	P<0.0001<br>P=0.7743<br>P=0.4058	Bonferroni
Pairwise Discrimination (PD)	2g	Sham: 12<br>Frac 20 cGy: 12	PD<br>Day of reversal: 1, 6, Last<br>Sham: 37.83, 65.16, 84.14<br>Frac 20 cGy: 41.89, 71.92, 88.28	Two-way ANOVA	interaction<br>day of testing<br>treatment	F (2, 42) = 43.04<br>F (1, 22) = 0.9363<br>F (2, 42) = 0.04335	P<0.0001<br>P=0.3438<br>P=0.9576	Bonferroni
Pairwise Discrimination (PD)	2h	Sham: 12<br>Frac 20 cGy: 12	PD<br>Day of reversal: 1, 6, Last<br>Sham: 18.75, 9.569, 5.25<br>Frac 20 cGy: 19.17, 9.601, 4.25	Two-way ANOVA	interaction<br>day of testing<br>treatment	F (2, 42) = 57.37<br>F (1, 22) = 0.01435<br>F (2, 42) = 0.1472	P<0.0001<br>P=0.9057<br>P=0.8636	Bonferroni
PD Reversal (Rev)	2i	Sham: 12<br>Frac 20 cGy: 12	Rev<br>Sham: Median: 15<br>Frac 20 cGy: Median: 14	Log-rank (Mantel-Cox) test	NA	NA	P=0.4781	NA
PD Reversal (Rev)	2j	Sham: 12<br>Frac 20 cGy: 12	Rev<br>Day of reversal: 1, 8, 12, Last<br>Sham: 1800 ±, 1742, 1444, 1146<br>Frac 20 cGy: 1800, 1687, 1553, 1076	Two-way ANOVA	interaction<br>day of testing<br>treatment	F (3, 61) = 66.30<br>F (1, 22) = 0.009466<br>F (3, 61) = 0.9977	P<0.0001<br>P=0.9234<br>P=0.4001	Bonferroni
PD Reversal (Rev)	2k	Sham: 12<br>Frac 20 cGy: 12	Rev<br>Day of reversal: 1, 8, 12, Last<br>Sham: 13.7, 46.33, 69.14, 86<br>Frac 20 cGy: 15.27, 47.31, 61.94, 88	Two-way ANOVA	interaction<br>day of testing<br>treatment	F (3, 61) = 86.05<br>F (1, 22) = 0.03111<br>F (3, 61) = 0.3662	P<0.0001<br>P=0.8616<br>P=0.7776	Bonferroni
PD Reversal (Rev)	2l	Sham: 12<br>Frac 20 cGy: 12	Rev<br>Day of reversal: 1, 8, 12, Last<br>Sham: 30.5, 16.83, 10.92, 4.25<br>Frac 20 cGy: 33, 17.25, 12.18, 3.75	Two-way ANOVA	interaction<br>day of testing<br>treatment	F (3, 61) = 140.4<br>F (1, 22) = 0.6191<br>F (3, 61) = 0.4176	P<0.0001<br>P=0.4398<br>P=0.741	Bonferroni
General Touchscreen Training w/three windows	3a	Sham: 12<br>Frac 20 cGy: 12	Training Stage: HAB, IT, MT, MI, PI<br>Sham: 1, 1.08, 4.08, 1, 14.5<br>Frac 20 cGy: 1, 1, 4, 1.083, 11.67	Two-way RM ANOVA	interaction<br>training stage<br>treatment<br>subject	F (4, 88) = 2.273<br>F (4, 88) = 156.1<br>F (1, 22) = 3.218<br>F (22, 88) = 0.7572	P=0.0677<br>P<0.0001<br>P=0.0866<br>P=0.7672	Bonferroni<br>**PI: P<0.01
PAL	3c	Sham: 12<br>Frac 20 cGy: 12	PAL<br>Day of testing: 1, 6, 11, 16, 21, 26<br>Sham: 1800, 1800, 1776, 1730, 1580, 1458<br>Frac 20 cGy: 1800, 1773, 1770, 1716, 1694, 1597	Two-way RM ANOVA	interaction<br>day of testing<br>treatment<br>subject	F (27, 594) = 1.470<br>F (27, 594) = 9.219<br>F (1, 22) = 0.9081<br>F (22, 594) = 13.18	P=0.0606<br>P<0.0001<br>P=0.3510<br>P<0.0001	Bonferroni<br>Day 25: *P<0.05
PAL	3d	Sham: 12<br>Frac 20 cGy: 12	PAL<br>Day of testing: 1, 6, 11, 16, 21, 26<br>Sham: 11.83, 14.5, 14.42, 18.58, 22.67, 22.92<br>Frac 20 cGy: 12.17, 15.5, 16.25, 17.08, 18.42, 20	Two-way RM ANOVA	interaction<br>day of testing<br>treatment<br>subject	F (27, 594) = 2.006<br>F (27, 594) = 18.39<br>F (1, 22) = 0.03083<br>F (22, 594) = 16.76	P=0.0021<br>P<0.0001<br>P=0.8622<br>P<0.0001	Bonferroni
PAL	3e	Sham: 12<br>Frac 20 cGy: 12	PAL<br>Day of testing: 1, 6, 11, 16, 21, 26<br>Sham: 40.61, 50.56, 49.19, 56.7, 63.61, 67.5<br>Frac 20 cGy: 47.34, 43.58, 44.17, 53.05, 50.25, 61.09	Two-way RM ANOVA	interaction<br>day of testing<br>treatment<br>subject	F (27, 594) = 1.383<br>F (27, 594) = 5.234<br>F (1, 22) = 3.853<br>F (22, 594) = 6.371	P=0.0956<br>P<0.0001<br>P=0.0624<br>P<0.0001	Bonferroni
PAL	3f	Sham: 12<br>Frac 20 cGy: 12	PAL<br>Day of testing: 1, 6, 11, 16, 21, 26<br>Sham: 22.08, 18.92, 20, 18.25, 15.08, 12.67<br>Frac 20 cGy: 23.5, 21.08, 20, 20.17, 16.5, 17	Two-way RM ANOVA	interaction<br>day of testing<br>treatment<br>subject	F (27, 594) = 0.8463<br>F (27, 594) = 4.188<br>F (1, 22) = 4.242<br>F (22, 594) = 6.810	P=0.6909<br>P<0.0001<br>P=0.0515<br>P<0.0001	Bonferroni
VMCL	3i	Sham: 11<br>Frac 20 cGy: 11	VMCL Training, VMCL<br>Sham: 10.18, 21.64<br>Frac 20 cGy: 9.273, 27	Two-way RM ANOVA	interaction<br>test type<br>treatment<br>subject	F (1, 20) = 1.697<br>F (1, 20) = 36.72<br>F (1, 20) = 0.5476<br>F (20, 20) = 1.563	P=0.2075<br>P<0.0001<br>P=0.4679<br>P=0.1632	Bonferroni
VMCL training	3j	Sham: 11<br>Frac 20 cGy: 11	VMCL training<br>Sham: Median: 10<br>Frac 20 cGy: Median: 9	Log-rank (Mantel-Cox) test	NA	NA	P=0.5295	NA
VMCL training	3k	Sham: 11<br>Frac 20 cGy: 11	VMCL training<br>Day of testing: 1, Last<br>Sham: 1604, 1515<br>Frac 20 cGy: 1607, 1460	Two-way RM ANOVA	interaction<br>day of testing<br>treatment<br>subject	F (1, 20) = 0.1484<br>F (1, 20) = 2.491<br>F (1, 20) = 0.1647<br>F (20, 20) = 0.7316	P=0.7041<br>P=0.1302<br>P=0.6892<br>P=0.7545	Bonferroni
VMCL training	3l	Sham: 11<br>Frac 20 cGy: 11	VMCL training<br>Day of testing: 1, Last<br>Sham: 22.45, 24.82<br>Frac 20 cGy: 22.09, 25	Two-way RM ANOVA	interaction<br>day of testing<br>treatment<br>subject	F (1, 20) = 0.07550<br>F (1, 20) = 7.055<br>F (1, 20) = 0.008803<br>F (20, 20) = 0.9530	P=0.7863<br>P=0.0152<br>P=0.9262<br>P=0.5423	Bonferroni
VMCL training	3m	Sham: 11<br>Frac 20 cGy: 11	VMCL training<br>Day of testing: 1, Last<br>Sham: 85.92, 87.58<br>Frac 20 cGy: 74.32, 78.73	Two-way RM ANOVA	interaction<br>day of testing<br>treatment<br>subject	F (1, 20) = 0.2182<br>F (1, 20) = 1.064<br>F (1, 20) = 1.803<br>F (20, 20) = 6.712	P=0.6454<br>P=0.3147<br>P=0.1944<br>P<0.0001	Bonferroni
VMCL training	3n	Sham: 11<br>Frac 20 cGy: 11	VMCL training<br>Day of testing: 1, Last<br>Sham: 85.92, 87.58<br>Frac 20 cGy: 74.32, 78.73	Two-way RM ANOVA	interaction<br>day of testing<br>treatment<br>subject	F (1, 20) = 0.2182<br>F (1, 20) = 1.064<br>F (1, 20) = 1.803<br>F (20, 20) = 6.712	P=0.6454<br>P=0.3147<br>P=0.1944<br>P<0.0001	Bonferroni
VMCL	3o	Sham: 11<br>Frac 20 cGy: 11	VMCL<br>Sham: Median: 22<br>Frac 20 cGy: Median: 23	Log-rank (Mantel-Cox) test	NA	NA	P=0.4313	NA
VMCL	3p	Sham: 11<br>Frac 20 cGy: 11	VMCL<br>Day of reversal: 1, 8, Last<br>Sham: 1800, 1800, 1592<br>Frac 20 cGy: 1800, 1800, 1691	Two-way RM ANOVA	interaction<br>day of testing<br>treatment<br>subject	F (2, 40) = 2.285<br>F (2, 40) = 23.85<br>F (1, 20) = 2.285<br>F (20, 40) = 1.000	P=0.1149<br>P<0.0001<br>P=0.1463<br>P=0.4827	Bonferroni<br>Last: *P<0.05
VMCL	3q	Sham: 11<br>Frac 20 cGy: 11	VMCL<br>Day of reversal: 1, 8, Last<br>Sham: 50.06, 56.33, 87.33<br>Frac 20 cGy: 39.96, 54.33, 89.52	Two-way RM ANOVA	interaction<br>day of testing<br>treatment<br>subject	F (2, 40) = 0.5196<br>F (2, 40) = 27.40<br>F (1, 20) = 0.8370<br>F (20, 40) = 0.5205	P=0.5987<br>P<0.0001<br>P=0.3711<br>P=0.9405	Bonferroni

VMCL	3r	Sham: 11	VMCL					Two-way RM ANOVA	interaction	F (2, 40) = 0.4245	P=0.6570	Bonferroni
			Day of reversal	1	8	Last			day of testing	F (2, 40) = 5.525	P=0.0076	
			Sham	26.07	24.5	11.98			treatment	F (1, 20) = 1.219	P=0.2826	
		Frac 20 cGy: 11	Frac 20 cGy	34.91	30.05	9.376			subject	F (20, 40) = 0.4638	P=0.9660	
VMCL	3s	Sham: 11	VMCL					Two-way RM ANOVA	interaction	F (2, 40) = 0.4920	P=0.6150	Bonferroni
			Day of reversal	1	8	Last			day of testing	F (2, 40) = 77.87	P<0.0001	
			Sham	22.91	15.82	9.091			treatment	F (1, 20) = 0.07031	P=0.7936	
		Frac 20 cGy: 11	Frac 20 cGy	23.27	16.09	7.364			subject	F (20, 40) = 1.988	P=0.0319	
LD training	4b	Sham: 12	LD training					Log-rank (Mantel-Cox) test	NA	NA	P=0.4820	NA
			Sham	Median: 2								
		Frac 20 cGy: 12	Frac 20 cGy	Median: 2								
LD training	4c	Sham: 12	LD training					Two-tailed t-test	NA	NA	P=0.3083	NA
			Sham	2.333 ± 0.2247								
		Frac 20 cGy: 12	Frac 20 cGy	2.083 ± 0.08333								
LD training	4d	Sham: 12	LD training					Two-tailed t-test	NA	NA	P=0.0055	NA
			Sham	1343 ± 87.70								
		Frac 20 cGy: 12	Frac 20 cGy	1035 ± 48.26								
LD training	4e	Sham: 12	LD training					Two-tailed t-test	NA	NA	P=0.6300	NA
			Sham	45.67 ± 4.074								
		Frac 20 cGy: 12	Frac 20 cGy	48.33 ± 3.633								
Location Discrimination (LD)	4g	Sham: 12	Location Discriminaton (LD)					Log-rank (Mantel-Cox) test	NA	NA	P=0.0216	NA
			Sham	Median: 6								
		Frac 20 cGy: 12	Frac 20 cGy	Median: 4								
Location Discrimination (LD)	4h	Sham: 12	Location Discriminaton (LD)					Two-tailed t-test	NA	NA	P=0.0312	NA
			Sham	6.083 ± 0.8657								
		Frac 20 cGy: 12	Frac 20 cGy	3.667 ± 0.5946								
Location Discrimination (LD)	4i	Sham: 12	Location Discriminaton (LD)					Two-tailed t-test	NA	NA	P=0.1688	NA
			Sham	1766 ± 21.22								
		Frac 20 cGy: 12	Frac 20 cGy	1706 ± 36.77								
Location Discrimination (LD)	4j	Sham: 12	Location Discriminaton (LD)					Two-way RM ANOVA	interaction	F (1, 22) = 0.3110	P=0.5827	Bonferroni
			Separation	Large	Small				separation	F (1, 22) = 0.07774	P=0.7830	
			Sham	24.08	24.33				treatment	F (1, 22) = 0.000	P>0.9999	
		Frac 20 cGy: 12	Frac 20 cGy	24.25	24.17				subject	F (22, 22) = 0.8445	P=0.6523	
Location Discrimination (LD)	4k	Sham: 12	Location Discriminaton (LD)					Two-tailed t-test	NA	NA	P=0.0045	NA
			Sham	36.31 ± 5.037								
		Frac 20 cGy: 12	Frac 20 cGy	61.66 ± 6.235								
Location Discrimination (LD)	4l	Sham: 12	Location Discriminaton (LD)					Two-way RM ANOVA	interaction	F (1, 22) = 0.02948	P=0.8652	Bonferroni Large/Small: *P<0.05
			Separation	Large	Small				separation	F (1, 22) = 6.460	P=0.0186	
			Sham	30.69	45.2				treatment	F (1, 22) = 9.477	P=0.0055	
		Frac 20 cGy: 12	Frac 20 cGy	55.58	68.26				subject	F (22, 22) = 2.121	P=0.0423	
Location Discrimination (LD)	4m	Sham: 12	Location Discriminaton (LD)					Two-tailed t-test	NA	NA	P=0.6237	NA
			Sham	142.2 ± 17.05								
		Frac 20 cGy: 12	Frac 20 cGy	153.4 ± 14.74								
Location Discrimination (LD)	4n	Sham: 12	Location Discriminaton (LD)					Two-tailed t-test	NA	NA	P=0.5804	NA
			Sham	1.306 ± 0.08350								
		Frac 20 cGy: 12	Frac 20 cGy	1.362 ± 0.05551								
Location Discrimination (LD)	4o	Sham: 12	Location Discriminaton (LD)					Two-tailed t-test	NA	NA	P=0.4245	NA
			Sham	6.368 ± 0.8784								
		Frac 20 cGy: 12	Frac 20 cGy	5.472 ± 0.6644								
Location Discrimination (LD)	4p	Sham: 12	Location Discriminaton (LD)					Two-tailed t-test	NA	NA	P=0.5118	NA
			Sham	5.683 ± 0.7225								
		Frac 20 cGy: 12	Frac 20 cGy	6.758 ± 1.441								
Location Discrimination (LD)	4q	Sham: 12	Location Discriminaton (LD)					Two-way RM ANOVA	interaction	F (3, 66) = 0.5372	P=0.6584	Bonferroni
			Block	1	2	3	4		block	F (3, 66) = 35.94	P<0.0001	
			Sham	489.4	320.9	307.7	358.4		treatment	F (1, 22) = 0.07455	P=0.7874	
		Frac 20 cGy: 12	Frac 20 cGy	502.6	292.5	322.2	342		subject	F (22, 66) = 1.150	P=0.3222	
Location Discrimination (LD)	4r	Sham: 12	Location Discriminaton (LD)					Two-way RM ANOVA	interaction	F (3, 66) = 1.173	P=0.3267	Bonferroni Last: **P<0.01
			Block	1	2	3	4		block	F (3, 66) = 1.923	P=0.1344	
			Sham	35.83	38.33	35	38.33		treatment	F (1, 22) = 8.146	P=0.0092	
		Frac 20 cGy: 12	Frac 20 cGy	55	55.83	55	71.67		subject	F (22, 66) = 5.478	P<0.0001	
Location Discrimination (LD)	4s	Sham: 12	Location Discriminaton (LD)					Two-way RM ANOVA	interaction	F (3, 66) = 1.056	P=0.3737	Bonferroni
			Block	1	2	3	4		block	F (3, 66) = 4.829	P=0.0042	
			Sham	34.83	30	31.5	27.75		treatment	F (1, 22) = 0.1750	P=0.6797	
		Frac 20 cGy: 12	Frac 20 cGy	43.83	30.75	30.25	26.67		subject	F (22, 66) = 3.529	P<0.0001	
Location Discrimination (LD)	4t	Sham: 12	Location Discriminaton (LD)					Two-way RM ANOVA	interaction	F (3, 66) = 1.010	P=0.3938	Bonferroni
			Block	1	2	3	4		block	F (3, 66) = 2.400	P=0.0756	
			Sham	6.833	11.92	8.917	8.667		treatment	F (1, 22) = 0.000	P>0.9999	
		Frac 20 cGy: 12	Frac 20 cGy	8.833	10	8	9.5		subject	F (22, 66) = 2.678	P=0.0011	
56Fe (CDFC)	5a	Sham: 10	Block	2	4	6	8	Two-way RM ANOVA	blocks	F (6, 54) = 3.106	P=0.0110	Bonferroni Block 5: ****P<0.0001 Block 6: ****P<0.0001 Block 7: ****P<0.0001 Block 8: **P<0.01
			Context A	28.71	37.43	43	40.29		context	F (1, 9) = 38.45	P=0.0002	
			Context B	25.43	28.86	25.57	25.71		interaction	F (6, 54) = 2.478	P=0.0344	
56Fe (CDFC)	5b	Frac 20 cGy: 10	Block	2	4	6	8	Two-way RM ANOVA	blocks	F (6, 54) = 0.8698	P=0.5232	Bonferroni Block 2: **P<0.01 Block 3: ***P<0.001 Block 4: **P<0.01 Block 5: ***P<0.001 Block 6: ****P<0.0001
			Context A	24.86	24.57	17.43	22		context	F (1, 9) = 137.8	P<0.0001	
			Context B	9.858	11.43	18	19.57		interaction	F (6, 54) = 1.584	P=0.1698	
56Fe (CDFC)	5c	Non-Frac 20 cGy: 9	Block	2	4	6	8	Two-way RM ANOVA	blocks	F (6, 48) = 2.239	P=0.0551	Bonferroni Block 2: **P<0.01 Block 3: **P<0.01 Block 4: ***P<0.001 Block 5: ****P<0.0001
			Context A	35.24	44.13	39.84	45.4		context	F (1, 8) = 31.30	P=0.0005	
			Context B	24.44	30.64	26.51	23.49		interaction	F (6, 48) = 3.031	P=0.0135	
56Fe Block 2 (CDFC)	5d	Sham: 10	Treatment	Sham	Frac 20 cGy	Non-Frac 20 cGy		Two-way RM ANOVA	interaction	F (2, 26) = 1.917	P=0.1673	Bonferroni Frac/Non-Frac: **P<0.01
		Frac 20 cGy: 10	Context A	28.71	34.71	35.24			treatment	F (2, 26) = 0.1356	P=0.8738	
		Non-Frac 20 cGy: 9	Context B	25.43	24.86	24.44			context	F (1, 26) = 21.62	P<0.0001	
									Subject	F (26, 26) = 8.519	P<0.0001	
56Fe Block 4 (CDFC)	5e	Sham: 10	Treatment	Sham	Frac 20 cGy	Non-Frac 20 cGy		Two-way RM ANOVA	interaction	F (2, 26) = 0.3829	P=0.6857	Bonferroni Frac: *P<0.05 Non-Frac: **P<0.01
		Frac 20 cGy: 10	Context A	37.43	36	44.13			treatment	F (2, 26) = 0.4833	P=0.6222	
		Non-Frac 20 cGy: 9	Context B	28.86	24.57	30.64			context	F (1, 26) = 23.70	P<0.0001	
									Subject	F (26, 26) = 6.533	P<0.0001	
56Fe Block 6 (CDFC)	5f	Sham: 10	Treatment	Sham	Frac 20 cGy	Non-Frac 20 cGy		Two-way RM ANOVA	interaction	F (2, 26) = 0.8509	P=0.4386	Bonferroni Sham/Frac: ****P<0.0001 Non-Frac: ***P<0.0002
		Frac 20 cGy: 10	Context A	43	35.43	39.84			treatment	F (2, 26) = 0.9621	P=0.3953	
		Non-Frac 20 cGy: 9	Context B	25.57	17.43	26.51			context	F (1, 26) = 107.7	P<0.0001	
									Subject	F (26, 26) = 10.49	P<0.0001	
28Si (CDFC)	6a	Sham: 8	Block	2	4	6	8	Two-way RM ANOVA	blocks	F (6, 42) = 4.152	P=0.0023	Bonferroni Block 5: **P<0.001 Block 8: ***P<0.0001
			Context A	40.18	55.89	49.11	57.86		context	F (1, 7) = 12.51	P=0.0095	
			Context B	37.68	46.61	40.89	38.39		interaction	F (6, 42) = 1.937	P=0.0970	
28Si (CDFC)	6b	20 cGy: 8	Block	2	4	6	8	Two-way RM ANOVA	blocks	F (6, 42) = 2.270	P=0.0548	Bonferroni Block 6: ****P<0.0001 Block 8: **P<0.01
			Context A	39.29	52.86	60.36	57.68		context	F (1, 7) = 9.797	P=0.0166	
			Context B	45.36	44.64	34.29	41.79		interaction	F (6, 42) = 4.779	P=0.0009	
28Si (CDFC)	6c	100 cGy: 8	Block	2	4	6	8	Two-way RM ANOVA	blocks	F (6, 42) = 2.777	P=0.0229	Bonferroni Block 3: *P<0.05 Block 4: **P<0.01 Block 5: *P<0.05 Block 6: *P<0.05
			Context A	36.07	51.07	48.57	51.25		context	F (1, 7) = 16.68	P=0.0047	
			Context B	33.39	36.79	36.43	38.75		interaction	F (6, 42) = 0.9790	P=0.4514	
28Si Block 2 (CDFC)	6d	Sham: 8	Treatment	Sham	20 cGy	100 cGy		Two-way RM ANOVA	interaction	F (2, 21) = 0.9076	P=0.4187	Bonferroni
		20 cGy: 8	Context A	40.18	39.29	36.07			treatment	F (2, 21) = 1.657	P=0.2147	
		100 cGy: 8	Context B	37.68	45.36	33.39			context	F (1, 21) = 0.009692	P=0.9225	
									Subject	F (21, 21) = 1.266	P=0.2968	

Col 1	Col 2	Col 3	Col 4	Col 5	Col 6	Col 7	Col 8	Col 9	Col 10	Col 11	Col 12	Col 13	Col 14
28Si Block 4 (CDFC)	6e	Sham: 8	Treatment	Sham	20 cGy	100 cGy			Two-way RM ANOVA	interaction	F (2, 21) = 0.6073	P=0.5541	Bonferroni<br>100 cGy:**P<0.01
		20 cGy: 8	Context A	55.89	52.86	51.07				treatment	F (2, 21) = 1.158	P=0.3334	
		100 cGy: 8	Context B	46.61	44.64	36.79				context	F (1, 21) = 19.48	P=0.0002	
										Subject	F (21, 21) = 2.767	P=0.0120	
28Si Block 6 (CDFC)	6f	Sham: 8	Treatment	Sham	20 cGy	100 cGy			Two-way RM ANOVA	interaction	F (2, 21) = 4.212	P=0.0290	Bonferroni<br>20 cGy: ****P<0.0001<br>100: *P<0.05
		20 cGy: 8	Context A	49.11	60.36	48.57				treatment	F (2, 21) = 0.3839	P=0.6859	
		100 cGy: 8	Context B	40.89	34.29	36.43				context	F (1, 21) = 34.37	P<0.0001	
										Subject	F (21, 21) = 2.897	P=0.0092	
DCX cell #	7b	Sham: 10<br>Frac 20 cGy: 10<br>Non-Frac 20 cGy: 9	Treatment	Sham	Frac 20 cGy	Non-Frac 20 cGy			One-way ANOVA	NA	F (2, 26) = 5.863	P=0.0079	Bonferroni<br>Sham vs Frac: *P<0.05<br>Sham vs Non-Frac: *P<0.05
				3942 ± 366.6	2825 ± 229.0	2614 ± 267.6							
DCX cell #	7c	Sham: 10	Bregma	-0.82	-1.54	-2.26	-2.98	-3.70	Two-way RM ANOVA	interaction	F (28, 364) = 1.260	P=0.1738	Bonferroni<br>Sham vs Non-Frac @ -1.78: *P<0.05<br>Sham vs Non-Frac @ -2.02: *P<0.05<br>Sham vs Frac @ -2.26: **P<0.01<br>Sham vs Non-Frac @ -3.46: *P<0.05<br>Sham vs Frac @ -3.70: **P<0.01<br>Sham vs Non-Frac @ -3.70: ***P<0.001
		Frac 20 cGy: 10	Sham	1.42E-14	29.6	55.5	48.5	33.70		bregma	F (14, 364) = 75.06	P<0.0001	
		Non-Frac 20 cGy: 9	Frac 20 cGy	0	23.7	35.7	35.9	14.30		treatment	F (2, 26) = 5.843	P=0.0080	
			Non-Frac 20 cGy	0	15.78	43.44	34.78	10.67		subject	F (26, 364) = 6.620	P<0.0001	

Table S2. Reporting statistical results of supplementary figures

Subject	Figure	n	Mean																Statistics (variables)	Main Effect	F Value	P value	Post hoc Test
Weights	S1a	Sham: 23	Month Post IRR	-2.5	-1.5	-0.5	0.5	1.5	2.5	3.5	4.5								Two-way RM ANOVA	Interaction	F (14, 609) = 0.7606	P=0.7125	Bonferroni
		Frac 20cGy: 42	Sham	29.44	33.49	35.22	34.08	35.69	36.95	40.3	42.1									Time	F (7, 609) = 547.5	P<0.0001	
		Non-Frac 20cGy: 25	Frac 20 cGy	28.77	31.94	33.74	32.25	34.14	36.2	39.14	40.6									Treatment	F (2, 87) = 1.495	P=0.2299	
			Non-Frac 20 cGy	28.82	32.04	33.64	33.07	34.23	36.04	39.28	40.8									Subjects	F (87, 609) = 31.21	P<0.0001	
Locomotor	S1b	Sham: 25	Time	6p	7p	8p	9p	10p	11p	12a	1a	2a	3a	4a	5a	6a	7a	8a	9a	Two-way RM ANOVA	Interaction	F (126, 5796) = 1.455	P=0.0008	Bonferroni 8p Sham vs Non-Frac: *P<0.05
		Frac 20cGy: 42	Sham	312.7	294	300	301	253	186	136	103	95.9	26.6	72.2	236	174	181	138	58.8		Time	F (63, 5796) = 109.2	P<0.0001	
			Frac 20 cGy	360.2	359	375	324	284	199	141	126	72.7	61.1	84.8	227	162	124	59.9	58.8		Treatment	F (2, 92) = 0.7391	P=0.4804	7a Sham vs Non-Frac vs Frac: **P<0.01
		Non-Frac 20cGy: 28	Non-Frac. 20 cGy	344.4	301	354	316	222	161	123	99.8	84.7	39.9	58.4	233	207	146	57.2	75.8		Subjects	F (92, 5796) = 13.93	P<0.0001	8a Sham vs Non-Frac vs Frac: *P<0.05
Time in Light (D/L)	S1c	Sham: 5<br>Frac 20cGy: 24<br>Non-Frac 20cGy: 8		Sham	Frac 20cGy	Non-Frac 20cGy													One-way ANOVA		F (2, 34) = 1.454	P=0.2477	NA
				140.3	127.8	173.4																	
Latency to Enter Light (D/L)	S1d	Sham: 5<br>Frac 20cGy: 24<br>Non-Frac 20cGy: 8		Sham	Frac 20cGy	Non-Frac 20cGy													One-way ANOVA		F (2, 32) = 2.900	P=0.0696	NA
				6.238	9.36	17.27																	
Flinch Response (PT)	S1e	Sham: 4<br>Frac 20cGy: 8<br>Non-Frac 20cGy: 8		Sham	Frac 20cGy	Non-Frac 20cGy													One-way ANOVA		F (2, 18) = 0.3061	P=0.7401	NA
				0.15	0.1563	0.1563																	
Vocalize Response (PT)	S1f	Sham: 4<br>Frac 20cGy: 8<br>Non-Frac 20cGy: 8		Sham	Frac 20cGy	Non-Frac 20cGy													One-way ANOVA		F (2, 18) = 0.05409	P=0.9475	NA
				0.3	0.3063	0.3																	
Jump Response (PT)	S1g	Sham: 4<br>Frac 20cGy: 8<br>Non-Frac 20cGy: 8		Sham	Frac 20cGy	Non-Frac 20cGy													One-way ANOVA		F (2, 18) = 6.468	P=0.0076	Bonferroni Frac vs Non-Frac: **P<0.01
				0.43	0.4625	0.4																	
Context (CFC)	S3c	Sham: 8<br>Frac 20cGy: 8<br>Non-Frac 20cGy: 8		Sham	Frac 20cGy	Non-Frac 20cGy													One-way ANOVA		F (2, 21) = 0.3035	P=0.7414	NA
				48.09	45.34	51.69																	
Cue (CFC)	S3d	Sham: 8		Sham	Frac 56Fe	Non-Frac 56Fe													Two-way RM ANOVA	Interaction	F (2, 21) = 0.8047	P=0.4605	Bonferroni
		Frac 20cGy: 8	Pre-Tone	25.36	21.79	21.07														Session	F (1, 21) = 110.2	P<0.0001	
		Non-Frac 20cGy: 8	During-Tone	71.79	58.21	69.65														Treatment	F (2, 21) = 1.514	P=0.2430	
																				Subjects	F (21, 21) = 0.9482	P=0.5479	
